# A novel mutation in the ATP7B gene causing hepatolenticular degeneration in a Chinese family: A case report

**DOI:** 10.1097/MD.0000000000038849

**Published:** 2024-08-02

**Authors:** Zhibo Zhou, Sainan Zhang, Yunjiao Bi, Wenyuan Duan, Hainv Gao

**Affiliations:** a Department of Infectious Diseases, Key Laboratory of Artificial Organs and Computational Medicine in Zhejiang Province, Shulan (Hangzhou) Hospital, Shulan International Medical College, Zhejiang Shuren University, Hangzhou, P. R. China; b Zhejiang Chinese Medical University, Hangzhou, P. R. China; c Department of Precision Medicine Testing Center, Key Laboratory of Artificial Organs and Computational Medicine in Zhejiang Province, Shulan (Hangzhou) Hospital, Shulan International Medical College, Zhejiang Shuren University, Hangzhou, P. R. China.

**Keywords:** *ATP7B*, case report, hepatolenticular degeneration, liver damage, mutation

## Abstract

**Introduction:**

Hepatolenticular degeneration (Wilson disease) is an autosomal recessive monogenic disorder caused by mutations in the ATPase copper transporting beta (*ATP7B*) gene located on human chromosome 13. This gene encodes a copper-transporting P-type ATPase (*ATP7B*). Recent studies have revealed that the *ATP7B* gene is predominantly affected by a few hotspot mutations, with the His1069Gln mutation in exon 14 accounting for 50 to 80% of cases. In China, the Arg778Leu mutation in exon 8 is the most prevalent. However, the discovery of novel mutant genes persists.

**Case presentation:**

A 56-year-old Chinese female was referred to our hospital with a liver injury and cirrhosis. Her parents, 2 younger brothers, and children exhibited no signs of liver function impairment. Whole-exome sequencing was conducted on the proband’s genomic DNA, and Sanger sequencing was performed on 6 family members for first-generation verification.

**Conclusions:**

We identified a novel c.3715G > T (p.Val1239Phe) variant mutation in the *ATP7B* gene in the patient. The *ATP7B* c.3715G > T (p.Val1239Phe) variant is predicted to impact the copper transport P-type ATPase. When combined with another mutant gene to form a compound heterozygous mutation, it can lead to hepatolenticular degeneration. This discovery broadens the range of pathogenic genes in the *ATP7B* gene.

## 1. Introduction

Wilson disease (WD) is a disorder of copper metabolism typically caused by genetic mutations. The onset age ranges from 3 to 30 years old, and it can affect multiple organs, resulting in complex clinical manifestations and diverse symptoms. Based on clinical presentations, the disease is primarily categorized into 3 types: liver type, brain type, and mixed type.^[1]^ When the liver is affected, clinical manifestations mainly include nausea, fatigue, and jaundice. In severe cases, liver cirrhosis and even liver failure may occur.^[2]^ When the central nervous system is involved, patients often experience symptoms such as abnormal behavior, personality changes, and ataxia.^[3]^ Hepatolenticular degeneration is closely linked to the ATPase copper transporting beta (*ATP7B*) gene, which primarily encodes P-type ATPase. The encoded protein specifically binds to the precursor of ceruloplasmin and forms ceruloplasmin. *ATP7B*, a known WD pathogenic gene, is situated on chromosome 13q14.3 and encodes a copper transporter P-type ATPase containing 1465 residues. This protein is composed of 6 metal-binding units, 8 transmembrane domains (TM), 1 actuator domain (A domain), a phosphorylation domain (P domain), and a nucleotide-binding domain (N domain).^[4]^ Over 700 mutations and approximately 800 single nucleotide polymorphisms (SNPs) have been identified in *ATP7B*, but only a few have been experimentally investigated.^[5]^ *ATP7B* is a large transmembrane protein, and its function and stability warrant further research.

Currently, the diagnosis of WD is primarily based on typical clinical manifestations, laboratory tests, and genetic testing. Treatment options include penicillamine, zinc preparations, and liver transplantation.^[6]^ WD is also one of the few neurogenetic diseases that can be treated effectively. The key to reducing mortality is early detection, diagnosis, and treatment. Early diagnosis and prompt treatment with the copper chelator D-penicillamine can significantly improve the clinical manifestations of WD patients, leading to life-long asymptomatic status. Initial studies suggested that WD is a rare disease, with a previously recognized incidence of 1/100,000 to 1/30,000 and a disease-causing gene carrier rate of approximately 1/90.^[7]^ Among the mutations, the 2333G > T (Arg778Leu) mutation in exon 8 has the highest frequency (48%), while the mutation frequency at the 2975C > T site in exon 13 is 29%.^[8]^ In this study, we identified a novel variant of *ATP7B* c.3715G > T (p.Val1239Phe) in a Chinese family. It is hypothesized that when this mutation occurs as a compound heterozygous or homozygous mutation, it may lead to hepatolenticular degeneration in affected family members.

## 2. Case presentation

### 2.1. General information

**Figure 1.**
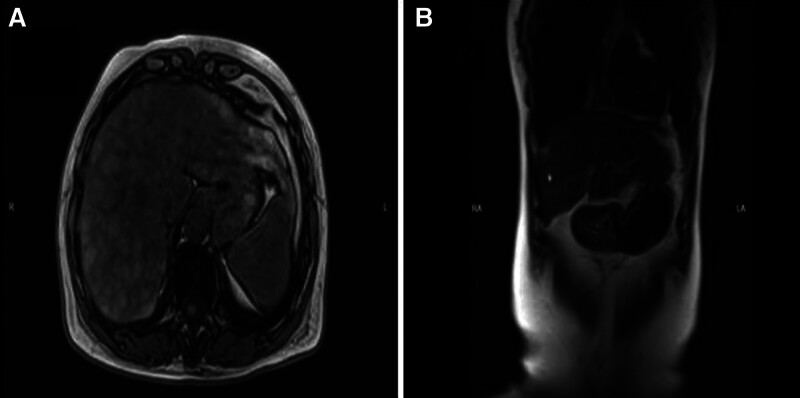
(a) Computed tomography image and (b) Magnetic resonance image show that liver profile was contoured like a wave. Imbalance between left and right liver lobes, widened liver fissure. Nodular liver cirrhosis was thought.

This study involves a three-generation Chinese family. The proband (II4) was a 56-year-old woman who had suffered from liver dysfunction (Table 1) and progressed to liver cirrhosis (Fig. 1). Her parents and other family members were unaffected. She is married and has a healthy child. This study was approved by the Medical Ethics Committee of Shulan (Hangzhou) Hospital, and each subject signed an informed consent form.

**Table 1:** Characteristics of the juvenile hemochromatosis patient.

Parameter(s)	Patient	Normal range
Age	56 year	
Sex	Female	
BMI	23 kg/m^2^	
Kaiser–Fleischers ring	Positive	
Albumin	28.7 g/L	40–55 g/L
Alanine aminotransferase	33 U/L	7–40 U/L
Aspartame aminotransferase	34 U/L	13–35 U/L
Y-glutamyltransferase	67 U/L	7–45 U/L
Alkaline phosphatase	217 U/L	4–150 U/L
Total bilirubin	18 umol/L	0–21 umol/L
Hyaluronic acid	411.30 ng/mL	0–100 ng/mL
Laminin	368.60 ng/mL	0–50 ng/Ml
Type III procollagen peptide	161.30 ng/mL	0–30 ng/mL
Type IV collagen	136ng/mL	0–30ng/ml
Ceruloplasmin	0.13g/L	0.2–0.6g/L

## 3. Methods

### 3.1. Liver function test

The patient had no history of medication, alcohol abuse, or red blood cell transfusions. There was no family history of chronic liver diseases, including hemochromatosis. Biochemical examinations revealed elevated γ-glutamyl transferase, alkaline phosphatase, and hyaluronic acid (Table 1). Tests for viral hepatitis and autoimmune liver diseases were negative. Liver function tests were also performed on 6 family members.

### 3.2. Genetic verification

**Figure 2.**
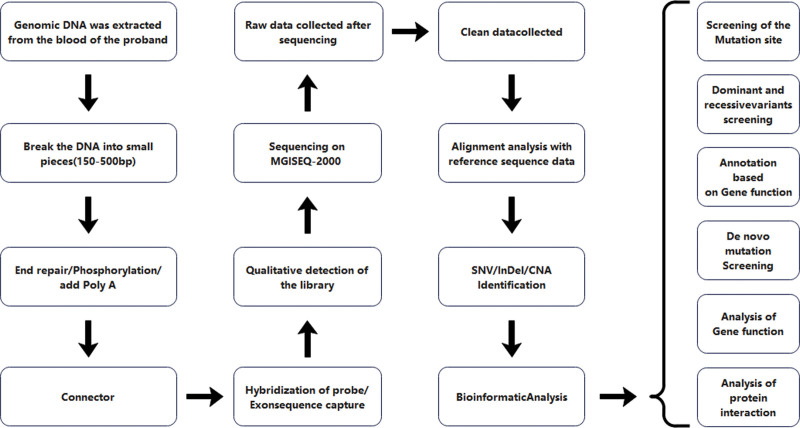
Schematic diagram of the detailed and comprehensive data interpretation process.

Genomic DNA was extracted from the proband’s blood samples using a MagPure Buffy Coat DNA Midi KF Kit (Magen) following the manufacturer’s instructions. The proband underwent whole-exome sequencing, with sequences captured using Allinone (Beijing Genomics Institute). The enriched library was then sequenced on a MGISEQ-2000 sequencer. The sequencing quality of the raw data was evaluated, and low-quality and contaminated reads from the connectors were removed. After that, the Burrows-Wheeler Aligner (BWA) software was used to align the sequences with the human reference genome Hg19, and the performance of sequence capture was tested. Single nucleotide variants (SNVs) and insertions and deletions (indels) were identified using the Genome Analysis Toolkit (GATK) software to generate nucleotide polymorphism results within the target regions. Variants identified by whole exome sequencing were filtered for data interpretation, focusing on those with a minor allele frequency <0.01 in databases such as dbSNP, HapMap, the 1000 Genomes Project, and an in-house database of 50,000 Chinese Han samples.^[9]^Data interpretation was performed based on the variant interpretation guidelines of the American College of Medical Genetics and Genomics (ACMG).^[10]^ Two deleterious variations were selected, and segregation analysis was performed (Fig. 2).

Venous blood samples were collected from all patients, and genomic DNA was isolated according to the method described by Ren et al.^[11]^ The coding exons and exon–intron boundaries of the ATP7B gene, from exons 1 to 21, were amplified using DNA from a precursor with corresponding primers (Table S1, Supplemental Digital Content, http://links.lww.com/MD/N230). PCR products were gel-purified (Weihansi Biomedical Technology Co., Ltd., Shanghai, China) and sequenced using BigDye 3.1 (Applied Biosystems, Foster City, CA). The raw data was directly sequenced using an ABI PRISM 3100 genetic analyzer and sequencing analysis software 5.3.1. The sequencing results were compared with the reported cDNA reference sequence (NM:000053).

### 3.3. Conservation of the replaced amino acid

We validated the mutation site in 100 vertebrates and did not find any changes (Table S2, Supplemental Digital Content, http://links.lww.com/MD/N231).

## 4. Results

### 4.1. Clinical analysis

We conducted an investigation and study on a three-generation family affected by hepatolenticular degeneration. Apart from the proband, who exhibited abnormal liver function and cirrhosis, all other family members demonstrated normal liver function.

### 4.2. Result verification

**Figure 3.**
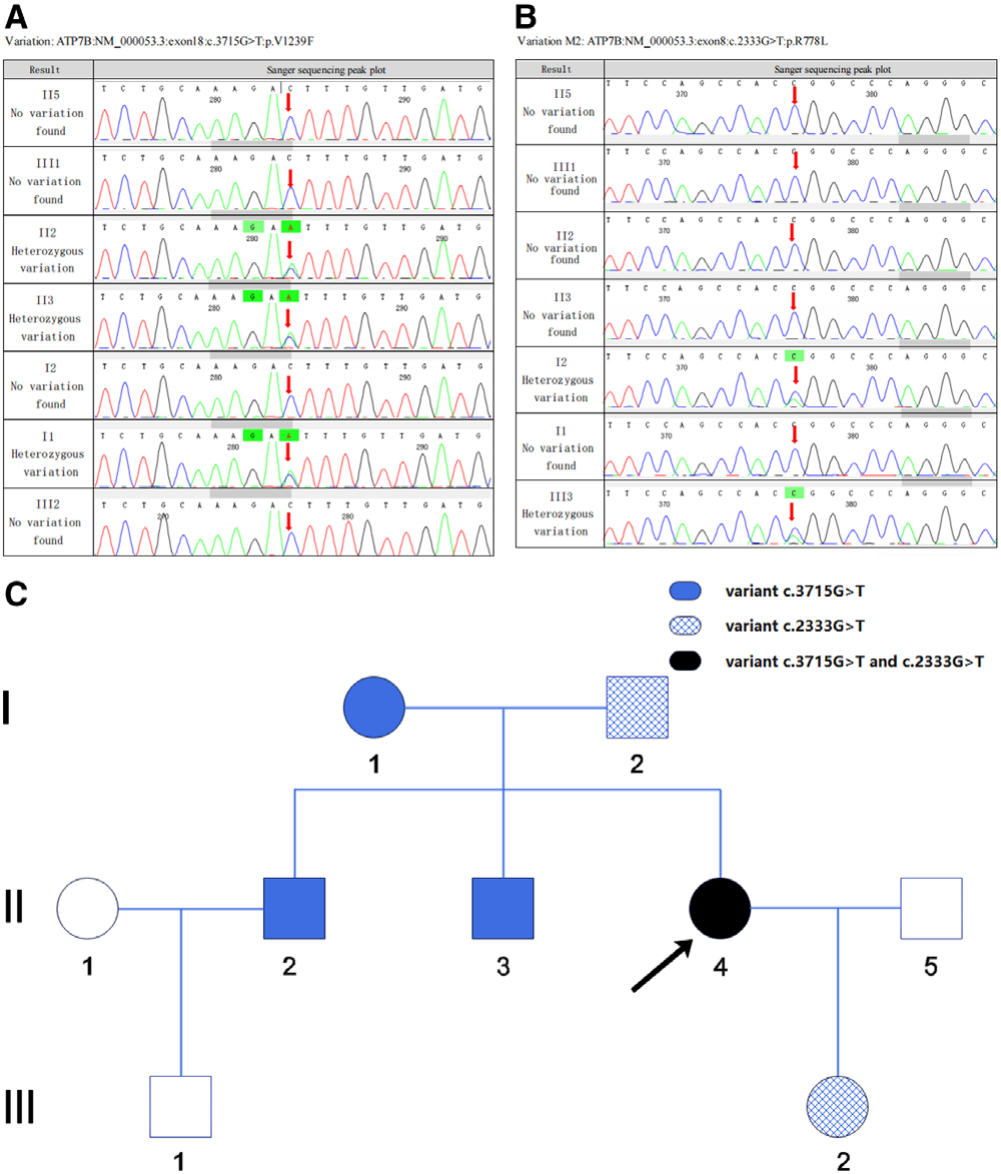
Identification of the pathogenic *ATP7B* variants in the proband’s family (a and b). Pedigree analyses to identify the family members with Wilson disease (c).

Sanger sequencing was performed on the DNA of 3 generations of family members. The results revealed that the proband’s parents each carried 1 of the 2 aforementioned gene mutations, while her siblings and children were all single heterozygous mutation carriers, and none of them had developed the disease. The proband, who carried 2 heterozygous mutations, had developed liver disease. The *ATP7B* c.3715G > T (p.Val1239Phe) variant was found to fulfill the criteria for an autosomal recessive genetic disease in the offspring of the parents, indicating that a homozygous variant or a compound heterozygous variant is required for disease manifestation (Fig. 3).

## 5. Discussion and conclusions

Through whole-exome sequencing, we discovered that a proband from a family in Ningbo, Zhejiang Province, carried 2 heterozygous mutations: the *ATP7B* c.3715G > T (p.Val1239Phe) variant on exon 18 and the *ATP7B* c.2333G > T (p.Arg778Leu) variant on exon 8. The proband exhibited manifestations of liver disease.

The pathogenicity of the *ATP7B* c.3715G > T (p.Val1239Phe) variant had not been previously verified. Through first-generation sequencing, we determined that the proband’s parents each carried 1 of the 2 gene mutations, while her siblings and children were all single heterozygous mutation carriers and did not develop the disease. The *ATP7B* c.3715G > T (p.Val1239Phe) variant was found to meet the requirements for an autosomal recessive genetic disease in the offspring of the parents, necessitating either a homozygous variant or a compound heterozygous variant. A review of relevant databases (https://www.ncbi.nlm.nih.gov and HGMD database) confirmed that the c.2333G > T missense mutation has been reported as pathogenic. In this case, we identified a novel mutation site for WD, with the patient carrying a heterozygous mutation in exon 18, c.3715G > T (p.Val1239Phe), resulting in an amino acid missense mutation.

Hepatolenticular degeneration is associated with various types of *ATP7B* gene mutations. Currently, the human gene mutation database includes more than 780 *ATP7B* gene mutations, comprising 491 missense/nonsense mutations, 65 splice site mutations, 187 small deletion/insertion mutations, and 12 large fragment deletions. Mutations can occur anywhere within the gene, including exons, introns, and even promoter regions.^[12]^ *ATP7B* gene mutations exhibit genetic differences across different races and regions. The most common mutation type in European populations is the His1069Gln mutation in exon 14, which is more prevalent in Italy, Sweden, and Romania, with an allele frequency of 30 to 70%. The clinical manifestations of this mutation primarily involve extrahepatic symptoms such as tremor, dystonia, and mental symptoms.^[13]^ In Asian populations, the most common mutation type is the Arg778Leu missense mutation in exon 8, which is most prevalent in China, South Korea, and Japan, with an allele frequency of 17.3% to 60%. The clinical symptoms associated with this mutation are predominantly liver damage, such as liver dysfunction and liver cirrhosis.^[14]^ A study of WD patients in China revealed that gene mutations in exons 8, 12, 13, and 16 accounted for more than 70% of cases. With scientific and technological advancements, an increasing number of novel WD gene mutations continue to be discovered, suggesting that the historical incidence of WD may have been seriously underestimated. For instance, a systematic review published in 2019 summarized global WD gene mutation research based on second-generation sequencing technology and estimated the global incidence of WD to be approximately 1/7194 (95% CI: 12.9–14.9).

In the present study, the proband had a late onset of the disease, with abnormal liver function manifesting in middle age and slowly progressing to liver cirrhosis. The whole-exome sequencing test revealed that the proband carried a compound heterozygous mutation rather than a homozygous mutation. We speculate that this may be related to the later age of onset, although further research is needed to confirm this hypothesis. WD is one of the few curable genetic diseases, and early diagnosis and treatment are of paramount importance. Timely intervention can prevent serious, irreversible tissue and organ damage, allowing affected individuals to achieve a life expectancy comparable to that of healthy individuals. Corneal pigment rings, ceruloplasmin content, and serum ceruloplasmin oxidase activity are all relatively specific diagnostic indicators. However, relying solely on clinical manifestations and biochemical examinations often leads to missed diagnoses and misdiagnoses, particularly in patients with liver disease. Genetic diagnosis is often an early and specific diagnostic method for the disease. It is crucial to conduct genetic testing on patients in a timely and accurate manner to facilitate patient rehabilitation and implement prenatal diagnosis to ensure the health of future generations. This study identified a novel pathogenic mutation in the *ATP7B* gene, expanding the *ATP7B* gene mutation map and providing a new basis for the molecular diagnosis of WD.

In conclusion, the present study identified a novel *ATP7B* c.3715G > T (p.Val1239Phe) variant in a Chinese family. This mutation is predicted to affect the copper transport P-type ATPase. When presented as a compound heterozygous mutation with another mutant gene, it can cause hepatolenticular degeneration. This finding enriches the *ATP7B* gene’s spectrum of pathogenic genes.

## Acknowledgments

First and foremost, I would like to show my deepest gratitude to my supervisor, Dr Hainv Gao, a respectable, responsible and resourceful scholar, who has provided me with valuable guidance in every stage of the writing of this article. I shall extend my thanks to our team for all their kindness and help. Last but not least, I’d like to thank all my friends and my family, for their encouragement and support.

## Author contributions

**Conceptualization:** Wenyuan Duan.

**Data curation:** Zhibo Zhou.

**Formal analysis:** Zhibo Zhou.

**Investigation:** Zhibo Zhou, Sainan Zhang, Yunjiao Bi.

**Writing – original draft:** Zhibo Zhou, Hainv Gao.

## Supplementary Material




