# Supplementary material for: A novel mutation in the ATP7B gene causing hepatolenticular degeneration in a Chinese family: A case report
Source: Medicine (Baltimore). 2024 Aug 2;103(31):e38849. doi: 10.1097/MD.0000000000038849 (PMC11296479; doi:10.1097/MD.0000000000038849)
Supplement: Supplementary file 1 [file medi-103-e38849-s001.pdf]

Site 1：NEW

chr13:52511800
rs185912036
NM_000053.3:c.3715G>T
>hg19_dna range=chr13:52511300-52512300 5'pad=0 3'pad=0 strand=+
repeatMasking=none
TAAAACGCCTCTAGCCAGCCAGTGAGTGAGCCACTCACTAACCCCAGCAG
GAACCTGGGAGACAGAAGCCTTTCTGGGCGCAGCTGGAGCACAGTGGGTA
AGAGCTGCCTACCTGCTGCAATGGGTATCCCAACCAGGTTATAAATCAGT
GCCAGGACCAGGTTGATGCGTATCCTTCGGACAGTCCTCTTGGAAAGGTG
AATGCTAGCCACCACATCCAGCAAATCATTCTGATGGAGAGGAGCACACA
GTGAGGAAGGGGTCTGCCCATTGCCCTCCCAGCACCCACAGCCTGGCTGC
AGCCACGCTCACTCTGATAAGGACGACGTCGGCTGCCTCGATGGCCACAT
CCGTGCCGGTGCCAATGGCCACACCCATGTCTGCCTGGGCCAAGGCCGGG
GAGTCATTGACCCCATCCCCCACCATGGCGACTTTCTTCCCTTTATTCTG
GAGCTCCTGGACCTTGGCCACCTTGTGCGAAGGCAGCACCTCTGCAAAGA
CTTTGTTGATGCCAACCTAAGACAAAAGGAAGGCAATGCCTAGTGTTGGC
AAAAGGTATCAGATAGCAGCAGAAACCTCAAGTTACCCTTGCCCCCTCTG
CCCTCGGCCTCTGGGTCCAGTCAGTGATGTTGGTCAACCACACCCTGCAG
GTTTGCTGTCACAGGGCCAGTTTATCCCTGGCTCCTGCCCCAACACCCTG
CACTGGTTTAGGCCCTGTCCTTTTCTGGATGTGCAAACAACCCCCTTGCT
GGTCTCCCTGTCCACGGCTCAGTCCTCCAACTGTCTTCTCCATGTGCTGC
CCCCATGGTCTTTCCAAAGCATCCGTCCGGCCATGTCCCTCCCTACTCTC
CTGCTTCACATCCAGTAGACCCCATGCATCTCCCAGAGGAGGGCAGCTCA
ACTCTTGGGTCTGCTTCCGAGGGTCCCAGAGTCCCAGGGTCCCAGGGTCT
GGCAGCTGGTGCCACACTTGACCATACCATTCAGCTCCCCGCGGCATCTC
C

>chr13:52511504+52512011 508bp CTAGCCACCACATCCAGCAA CCTAAACCAGTGCAGGGTGT
CTAGCCACCACATCCAGCAAatcattctgatggagaggagcacacagtga
ggaaggggtctgcccattgccctcccagcacccacagcctggctgcagcc
acgctcactctgataaggacgacgtcggctgcctcgatggccacatccgt
gccggtgccaatggccacacccatgtctgcctgggccaaggccggggagt
cattgaccccatcccccaccatggcgactttcttccctttattctggagc
tcctggaccttggccaccttgtgcgaaggcagcacctctgcaaagacttt
gttgatgccaacctaagacaaaaggaaggcaatgcctagtgttggcaaaa
ggtatcagatagcagcagaaacctcaagttacccttgcccccctctgccct
cggcctctgggtccagtcagtgatgttggtcaaccacaccctgcaggttt
gctgtcacagggccagtttatccctggctcctgccccaACACCCTGCACT
GGTTTAGG

Site 2：

chr13:52532469
rs28942074
NM_000053.4（ATP7B）：c.2333G> T（p.Arg778Leu）

>hg19_dna range=chr13:52531969-52532969 5'pad=0 3'pad=0 strand=+ repeatMasking=none
CATTTCAGACTGGAAACAAACATCAGTCTTCCCACTGACCATTTTCTTTT
AGGTTCAATCTAATTTCTTTTAGGGCCTGAGTTCCATTCTCTTGTAAGAT
ACATTTCAGTGTTGGGGACAATGAAGGGTGTTAAAAATGGCATGACCTTG
AGGAGGATGTATGGATGTCTAGACCAACTACATATTCAGTTTTGCACCAA
GAGACAATGTAGGCTCTGCCCTGAAGGCCAGGTTTCTTTAGTTTACACAA
TACACCTGAATGATGGTTTTAATAATTAACCAGATTAGCTGGGATTTCAG
AAGTAGTGACCAATTTGGAGATTAGTGACTAGAGCACCTTAATTATATGG
AGGTTTCCTATTTCTTTAAGTCTGTCTCTATGCTGTGTATAATTAGTAAT
TCTAAACATGGTGTTCAGAGGAAGTGAGATTTGTTTACTGAAGGAGCAGC
TCTTTTCTGAACCTGAAGCTGCTGTTACCTTTGCCAAGTGTTCCAGCCAC
CGGCCCAGGGCAATGAACACAAAGAGCATGGGGGGCGTGTCGAAGAATGT
CACAGGGCTCCTCTCCGCCTTCTCAGCCACAGCAACCACCAGGATGACCA
GAGAATAAACATAAGCAATGCTTGTGGCCAGGACGATGAGCACGTCCATG
TTGGCTGACCTGTGTCTCAGAGATTTGTAGGCCTGAACGTAGAAGTACCA
CCCACCGAGGAGCTGAAAGACAAGGACAGTGAAGGCTGCCAGCAAGTAGG
GAGGAGAGTTCAATGAGCGACACAGGGCCAAGGGCCCTGGGGATGGCAAA
GCCTCTAGCTTTGTGCACAGTCGTGACAGTACTTCTTCCTCTGTGATGGG
CGTTTATGAAATATACTTTCCCATTCAGAGGACTACTGTCCACAGGAATG
GATGACCAAGAGCATGTGAGACCTTTAAAACCCAGTGCTTACAACACAAA
TGTCCTCTAATAAGAGAATGGTGAAAAGTTGGGGAAAAAAATCACGACTC
A

>chr13:52532081+52532620 540bp TGGGGACAATGAAGGGTGTT
AACATGGACGTGCTCATCGT
TGGGGACAATGAAGGGTGTTaaaaatggcatgaccttgaggaggatgtat
ggatgtctagaccaactacatattcagttttgcaccaagagacaatgtag
gctctgccctgaaggccaggtttctttagtttacacaatacacctgaatg
atggttttaataattaaccagattagctgggatttcagaagtagtgacca
atttggagattagtgactagagcaccttaattatatggaggtttcctatt
tctttaagtctgtctctatgctgtgtataattagtaattctaaacatggt
gttcagaggaagtgagatttgtttactgaaggagcagctcttttctgaac
ctgaagctgctgttacctttgccaagtgttccagccaccggcccagggca
atgaacacaaagagcatggggggcgtgtcgaagaatgtcacagggctcct
ctccgccttctcagccacagcaaccaccaggatgaccagagaataaacat
aagcaatgcttgtggccaggACGATGAGCACGTCCATGTT
