# Supplementary material for: A novel mutation in the ATP7B gene causing hepatolenticular degeneration in a Chinese family: A case report
Source: Medicine (Baltimore). 2024 Aug 2;103(31):e38849. doi: 10.1097/MD.0000000000038849 (PMC11296479; doi:10.1097/MD.0000000000038849)
Supplement: Supplementary file 2 [file medi-103-e38849-s002.docx]

- [Home](https://genome.ucsc.edu/index.html)
- [Genomes](https://genome.ucsc.edu/cgi-bin/hgGateway?hgsid=2306903998_81N7R1Zas6CL9LKWAfmjniqz8ti7)
- [Genome Browser](https://genome.ucsc.edu/cgi-bin/hgTracks?hgsid=2306903998_81N7R1Zas6CL9LKWAfmjniqz8ti7)
- Tools
- Mirrors
- Downloads
- My Data
- Projects
- Help
- About Us

| \| \| **hg19 Human: Multiz Alignments of 100 Vertebrates**   \|  \| \| --- \| \|  \| Top of Form  Capitalize    exons based on                                                                                                  show    bases  Bottom of Form  Place cursor over species for alignment detail. Click on 'B' to link to browser for aligned species, click on 'D' to get DNA for aligned species.  **Alignment block 1 of 1 in window, 52511800 - 52511800, 1 bps**  [B](https://genome.ucsc.edu/cgi-bin/hgTracks?db=hg19&ct=&position=chr13%3A52511800-52511800) [D](https://genome.ucsc.edu/cgi-bin/hgc?o=52511799&g=getDna&i=chr13&c=chr13&l=52511799&r=52511800&db=hg19) Human c  [B](https://genome.ucsc.edu/cgi-bin/hgTracks?db=panTro4&ct=&position=chr13%3A51675321-51675321) [D](https://genome.ucsc.edu/cgi-bin/hgc?o=51675320&g=getDna&i=chr13&c=chr13&l=51675320&r=51675321&db=panTro4) Chimp c  [B](https://genome.ucsc.edu/cgi-bin/hgTracks?db=gorGor3&ct=&position=chr13%3A34231839-34231839) [D](https://genome.ucsc.edu/cgi-bin/hgc?o=34231838&g=getDna&i=chr13&c=chr13&l=34231838&r=34231839&db=gorGor3) Gorilla c  [B](https://genome.ucsc.edu/cgi-bin/hgTracks?db=ponAbe2&ct=&position=chr13%3A52260267-52260267) [D](https://genome.ucsc.edu/cgi-bin/hgc?o=52260266&g=getDna&i=chr13&c=chr13&l=52260266&r=52260267&db=ponAbe2) Orangutan c  [B](https://genome.ucsc.edu/cgi-bin/hgTracks?db=nomLeu3&ct=&position=chr5%3A79634456-79634456) [D](https://genome.ucsc.edu/cgi-bin/hgc?o=79634455&g=getDna&i=chr5&c=chr5&l=79634455&r=79634456&db=nomLeu3) Gibbon c  [B](https://genome.ucsc.edu/cgi-bin/hgTracks?db=rheMac3&ct=&position=chr17%3A31027728-31027728) [D](https://genome.ucsc.edu/cgi-bin/hgc?o=31027727&g=getDna&i=chr17&c=chr17&l=31027727&r=31027728&db=rheMac3) Rhesus c  [B](https://genome.ucsc.edu/cgi-bin/hgTracks?db=macFas5&ct=&position=chr17%3A31190383-31190383) [D](https://genome.ucsc.edu/cgi-bin/hgc?o=31190382&g=getDna&i=chr17&c=chr17&l=31190382&r=31190383&db=macFas5) Crab-eating macaque c  [B](https://genome.ucsc.edu/cgi-bin/hgTracks?db=papHam1&ct=&position=scaffold12103%3A26447-26447) [D](https://genome.ucsc.edu/cgi-bin/hgc?o=26446&g=getDna&i=scaffold12103&c=scaffold12103&l=26446&r=26447&db=papHam1) Baboon c  Green monkey c  [B](https://genome.ucsc.edu/cgi-bin/hgTracks?db=calJac3&ct=&position=chr1%3A62792102-62792102) [D](https://genome.ucsc.edu/cgi-bin/hgc?o=62792101&g=getDna&i=chr1&c=chr1&l=62792101&r=62792102&db=calJac3) Marmoset c  [B](https://genome.ucsc.edu/cgi-bin/hgTracks?db=saiBol1&ct=&position=JH378126%3A1355286-1355286) [D](https://genome.ucsc.edu/cgi-bin/hgc?o=1355285&g=getDna&i=JH378126&c=JH378126&l=1355285&r=1355286&db=saiBol1) Squirrel monkey c  [B](https://genome.ucsc.edu/cgi-bin/hgTracks?db=otoGar3&ct=&position=GL873740%3A433518-433518) [D](https://genome.ucsc.edu/cgi-bin/hgc?o=433517&g=getDna&i=GL873740&c=GL873740&l=433517&r=433518&db=otoGar3) Bushbaby c  Chinese tree shrew c  [B](https://genome.ucsc.edu/cgi-bin/hgTracks?db=speTri2&ct=&position=JH393375%3A4698063-4698063) [D](https://genome.ucsc.edu/cgi-bin/hgc?o=4698062&g=getDna&i=JH393375&c=JH393375&l=4698062&r=4698063&db=speTri2) Squirrel c  Lesser Egyptian jerboa c  Prairie vole c  [B](https://genome.ucsc.edu/cgi-bin/hgTracks?db=criGri1&ct=&position=KE381473%3A52151-52151) [D](https://genome.ucsc.edu/cgi-bin/hgc?o=52150&g=getDna&i=KE381473&c=KE381473&l=52150&r=52151&db=criGri1) Chinese hamster c  Golden hamster c  [B](https://genome.ucsc.edu/cgi-bin/hgTracks?db=mm10&ct=&position=chr8%3A21997671-21997671) [D](https://genome.ucsc.edu/cgi-bin/hgc?o=21997670&g=getDna&i=chr8&c=chr8&l=21997670&r=21997671&db=mm10) Mouse c  [B](https://genome.ucsc.edu/cgi-bin/hgTracks?db=rn5&ct=&position=chr16%3A74570598-74570598) [D](https://genome.ucsc.edu/cgi-bin/hgc?o=74570597&g=getDna&i=chr16&c=chr16&l=74570597&r=74570598&db=rn5&hgSeq.revComp=on) Rat c  [B](https://genome.ucsc.edu/cgi-bin/hgTracks?db=hetGla2&ct=&position=JH602061%3A2178453-2178453) [D](https://genome.ucsc.edu/cgi-bin/hgc?o=2178452&g=getDna&i=JH602061&c=JH602061&l=2178452&r=2178453&db=hetGla2&hgSeq.revComp=on) Naked mole-rat c  [B](https://genome.ucsc.edu/cgi-bin/hgTracks?db=cavPor3&ct=&position=scaffold_6%3A42185536-42185536) [D](https://genome.ucsc.edu/cgi-bin/hgc?o=42185535&g=getDna&i=scaffold_6&c=scaffold_6&l=42185535&r=42185536&db=cavPor3&hgSeq.revComp=on) Guinea pig c  Chinchilla c  Brush-tailed rat c  [B](https://genome.ucsc.edu/cgi-bin/hgTracks?db=oryCun2&ct=&position=chrUn0007%3A5712296-5712296) [D](https://genome.ucsc.edu/cgi-bin/hgc?o=5712295&g=getDna&i=chrUn0007&c=chrUn0007&l=5712295&r=5712296&db=oryCun2) Rabbit c  [B](https://genome.ucsc.edu/cgi-bin/hgTracks?db=ochPri3&ct=&position=JH802062%3A54766347-54766347) [D](https://genome.ucsc.edu/cgi-bin/hgc?o=54766346&g=getDna&i=JH802062&c=JH802062&l=54766346&r=54766347&db=ochPri3) Pika c  [B](https://genome.ucsc.edu/cgi-bin/hgTracks?db=vicPac2&ct=&position=KB632540%3A3871154-3871154) [D](https://genome.ucsc.edu/cgi-bin/hgc?o=3871153&g=getDna&i=KB632540&c=KB632540&l=3871153&r=3871154&db=vicPac2&hgSeq.revComp=on) Alpaca c  Bactrian camel c  [B](https://genome.ucsc.edu/cgi-bin/hgTracks?db=turTru2&ct=&position=JH494002%3A8602-8602) [D](https://genome.ucsc.edu/cgi-bin/hgc?o=8601&g=getDna&i=JH494002&c=JH494002&l=8601&r=8602&db=turTru2) Dolphin c  Killer whale c  Tibetan antelope c  [B](https://genome.ucsc.edu/cgi-bin/hgTracks?db=bosTau7&ct=&position=chr12%3A20757847-20757847) [D](https://genome.ucsc.edu/cgi-bin/hgc?o=20757846&g=getDna&i=chr12&c=chr12&l=20757846&r=20757847&db=bosTau7) Cow c  [B](https://genome.ucsc.edu/cgi-bin/hgTracks?db=oviAri3&ct=&position=chr10%3A21659453-21659453) [D](https://genome.ucsc.edu/cgi-bin/hgc?o=21659452&g=getDna&i=chr10&c=chr10&l=21659452&r=21659453&db=oviAri3) Sheep c  Domestic goat c  [B](https://genome.ucsc.edu/cgi-bin/hgTracks?db=equCab2&ct=&position=chr17%3A19331364-19331364) [D](https://genome.ucsc.edu/cgi-bin/hgc?o=19331363&g=getDna&i=chr17&c=chr17&l=19331363&r=19331364&db=equCab2&hgSeq.revComp=on) Horse c  [B](https://genome.ucsc.edu/cgi-bin/hgTracks?db=cerSim1&ct=&position=JH767768%3A19044349-19044349) [D](https://genome.ucsc.edu/cgi-bin/hgc?o=19044348&g=getDna&i=JH767768&c=JH767768&l=19044348&r=19044349&db=cerSim1) White rhinoceros c  [B](https://genome.ucsc.edu/cgi-bin/hgTracks?db=felCat5&ct=&position=chrA1%3A19368102-19368102) [D](https://genome.ucsc.edu/cgi-bin/hgc?o=19368101&g=getDna&i=chrA1&c=chrA1&l=19368101&r=19368102&db=felCat5&hgSeq.revComp=on) Cat c  [B](https://genome.ucsc.edu/cgi-bin/hgTracks?db=canFam3&ct=&position=chr22%3A223497-223497) [D](https://genome.ucsc.edu/cgi-bin/hgc?o=223496&g=getDna&i=chr22&c=chr22&l=223496&r=223497&db=canFam3&hgSeq.revComp=on) Dog c  [B](https://genome.ucsc.edu/cgi-bin/hgTracks?db=musFur1&ct=&position=GL897107%3A229047-229047) [D](https://genome.ucsc.edu/cgi-bin/hgc?o=229046&g=getDna&i=GL897107&c=GL897107&l=229046&r=229047&db=musFur1&hgSeq.revComp=on) Ferret c  [B](https://genome.ucsc.edu/cgi-bin/hgTracks?db=ailMel1&ct=&position=GL192556.1%3A1090364-1090364) [D](https://genome.ucsc.edu/cgi-bin/hgc?o=1090363&g=getDna&i=GL192556.1&c=GL192556.1&l=1090363&r=1090364&db=ailMel1&hgSeq.revComp=on) Panda c  Pacific walrus c  Weddell seal c  Black flying-fox c  [B](https://genome.ucsc.edu/cgi-bin/hgTracks?db=pteVam1&ct=&position=scaffold_6802%3A12535-12535) [D](https://genome.ucsc.edu/cgi-bin/hgc?o=12534&g=getDna&i=scaffold_6802&c=scaffold_6802&l=12534&r=12535&db=pteVam1&hgSeq.revComp=on) Megabat c  David's myotis (bat) c  [D](https://genome.ucsc.edu/cgi-bin/hgc?o=10844432&g=getDna&i=GL429774&c=GL429774&l=10844432&r=10844433&db=myoLuc2) Little brown bat c  Big brown bat c  [B](https://genome.ucsc.edu/cgi-bin/hgTracks?db=eriEur2&ct=&position=JH835440%3A1735734-1735734) [D](https://genome.ucsc.edu/cgi-bin/hgc?o=1735733&g=getDna&i=JH835440&c=JH835440&l=1735733&r=1735734&db=eriEur2) Hedgehog c  [B](https://genome.ucsc.edu/cgi-bin/hgTracks?db=sorAra2&ct=&position=JH798287%3A94760-94760) [D](https://genome.ucsc.edu/cgi-bin/hgc?o=94759&g=getDna&i=JH798287&c=JH798287&l=94759&r=94760&db=sorAra2) Shrew c  Star-nosed mole c  [B](https://genome.ucsc.edu/cgi-bin/hgTracks?db=loxAfr3&ct=&position=scaffold_23%3A34546033-34546033) [D](https://genome.ucsc.edu/cgi-bin/hgc?o=34546032&g=getDna&i=scaffold_23&c=scaffold_23&l=34546032&r=34546033&db=loxAfr3) Elephant c  Cape elephant shrew c  [B](https://genome.ucsc.edu/cgi-bin/hgTracks?db=triMan1&ct=&position=JH594650%3A337085-337085) [D](https://genome.ucsc.edu/cgi-bin/hgc?o=337084&g=getDna&i=JH594650&c=JH594650&l=337084&r=337085&db=triMan1&hgSeq.revComp=on) Manatee c  Cape golden mole c  [B](https://genome.ucsc.edu/cgi-bin/hgTracks?db=echTel2&ct=&position=JH980344%3A41059-41059) [D](https://genome.ucsc.edu/cgi-bin/hgc?o=41058&g=getDna&i=JH980344&c=JH980344&l=41058&r=41059&db=echTel2) Tenrec c  Aardvark c  [B](https://genome.ucsc.edu/cgi-bin/hgTracks?db=dasNov3&ct=&position=JH563071%3A91738-91738) [D](https://genome.ucsc.edu/cgi-bin/hgc?o=91737&g=getDna&i=JH563071&c=JH563071&l=91737&r=91738&db=dasNov3&hgSeq.revComp=on) Armadillo c  [B](https://genome.ucsc.edu/cgi-bin/hgTracks?db=monDom5&ct=&position=chr4%3A315667216-315667216) [D](https://genome.ucsc.edu/cgi-bin/hgc?o=315667215&g=getDna&i=chr4&c=chr4&l=315667215&r=315667216&db=monDom5&hgSeq.revComp=on) Opossum c  [B](https://genome.ucsc.edu/cgi-bin/hgTracks?db=sarHar1&ct=&position=chr3_GL849711_random%3A957167-957167) [D](https://genome.ucsc.edu/cgi-bin/hgc?o=957166&g=getDna&i=chr3_GL849711_random&c=chr3_GL849711_random&l=957166&r=957167&db=sarHar1&hgSeq.revComp=on) Tasmanian devil c  [D](https://genome.ucsc.edu/cgi-bin/hgc?o=1749014&g=getDna&i=KB398245&c=KB398245&l=1749014&r=1749015&db=falChe1) Saker falcon c  [D](https://genome.ucsc.edu/cgi-bin/hgc?o=1678931&g=getDna&i=KB391030&c=KB391030&l=1678931&r=1678932&db=falPer1&hgSeq.revComp=on) Peregrine falcon c  [D](https://genome.ucsc.edu/cgi-bin/hgc?o=67153633&g=getDna&i=chr1&c=chr1&l=67153633&r=67153634&db=ficAlb2) Collared flycatcher c  [D](https://genome.ucsc.edu/cgi-bin/hgc?o=2377585&g=getDna&i=KB913047&c=KB913047&l=2377585&r=2377586&db=zonAlb1) White-throated sparrow c  [B](https://genome.ucsc.edu/cgi-bin/hgTracks?db=geoFor1&ct=&position=JH739912%3A5692048-5692048) [D](https://genome.ucsc.edu/cgi-bin/hgc?o=5692047&g=getDna&i=JH739912&c=JH739912&l=5692047&r=5692048&db=geoFor1) Medium ground finch c  [B](https://genome.ucsc.edu/cgi-bin/hgTracks?db=taeGut2&ct=&position=chr1%3A55034579-55034579) [D](https://genome.ucsc.edu/cgi-bin/hgc?o=55034578&g=getDna&i=chr1&c=chr1&l=55034578&r=55034579&db=taeGut2&hgSeq.revComp=on) Zebra finch c  Tibetan ground jay c  [B](https://genome.ucsc.edu/cgi-bin/hgTracks?db=melUnd1&ct=&position=JH556585%3A4756815-4756815) [D](https://genome.ucsc.edu/cgi-bin/hgc?o=4756814&g=getDna&i=JH556585&c=JH556585&l=4756814&r=4756815&db=melUnd1&hgSeq.revComp=on) Budgerigar c  [D](https://genome.ucsc.edu/cgi-bin/hgc?o=23441&g=getDna&i=KB272330&c=KB272330&l=23441&r=23442&db=amaVit1&hgSeq.revComp=on) Parrot c  [D](https://genome.ucsc.edu/cgi-bin/hgc?o=9720&g=getDna&i=KE033471&c=KE033471&l=9720&r=9721&db=araMac1) Scarlet macaw c  [D](https://genome.ucsc.edu/cgi-bin/hgc?o=16142501&g=getDna&i=KB375372&c=KB375372&l=16142501&r=16142502&db=colLiv1) Rock pigeon c  [D](https://genome.ucsc.edu/cgi-bin/hgc?o=557672&g=getDna&i=KB742740&c=KB742740&l=557672&r=557673&db=anaPla1&hgSeq.revComp=on) Mallard duck c  [B](https://genome.ucsc.edu/cgi-bin/hgTracks?db=galGal4&ct=&position=chr1%3A169619531-169619531) [D](https://genome.ucsc.edu/cgi-bin/hgc?o=169619530&g=getDna&i=chr1&c=chr1&l=169619530&r=169619531&db=galGal4) Chicken c  [B](https://genome.ucsc.edu/cgi-bin/hgTracks?db=melGal1&ct=&position=chr1%3A172235552-172235552) [D](https://genome.ucsc.edu/cgi-bin/hgc?o=172235551&g=getDna&i=chr1&c=chr1&l=172235551&r=172235552&db=melGal1&hgSeq.revComp=on) Turkey c  [B](https://genome.ucsc.edu/cgi-bin/hgTracks?db=allMis1&ct=&position=JH731763%3A615080-615080) [D](https://genome.ucsc.edu/cgi-bin/hgc?o=615079&g=getDna&i=JH731763&c=JH731763&l=615079&r=615080&db=allMis1) American alligator c  [D](https://genome.ucsc.edu/cgi-bin/hgc?o=1107571&g=getDna&i=KB565453&c=KB565453&l=1107571&r=1107572&db=cheMyd1&hgSeq.revComp=on) Green seaturtle c  [B](https://genome.ucsc.edu/cgi-bin/hgTracks?db=chrPic1&ct=&position=JH584789%3A3461364-3461364) [D](https://genome.ucsc.edu/cgi-bin/hgc?o=3461363&g=getDna&i=JH584789&c=JH584789&l=3461363&r=3461364&db=chrPic1&hgSeq.revComp=on) Painted turtle c  [D](https://genome.ucsc.edu/cgi-bin/hgc?o=716743&g=getDna&i=JH209282&c=JH209282&l=716743&r=716744&db=pelSin1) Chinese softshell turtle c  [D](https://genome.ucsc.edu/cgi-bin/hgc?o=3761706&g=getDna&i=KB930164&c=KB930164&l=3761706&r=3761707&db=apaSpi1&hgSeq.revComp=on) Spiny softshell turtle c  [B](https://genome.ucsc.edu/cgi-bin/hgTracks?db=anoCar2&ct=&position=chr1%3A132840349-132840349) [D](https://genome.ucsc.edu/cgi-bin/hgc?o=132840348&g=getDna&i=chr1&c=chr1&l=132840348&r=132840349&db=anoCar2&hgSeq.revComp=on) Lizard c  [B](https://genome.ucsc.edu/cgi-bin/hgTracks?db=xenTro7&ct=&position=KB021650%3A94154507-94154507) [D](https://genome.ucsc.edu/cgi-bin/hgc?o=94154506&g=getDna&i=KB021650&c=KB021650&l=94154506&r=94154507&db=xenTro7) X. tropicalis c  [B](https://genome.ucsc.edu/cgi-bin/hgTracks?db=latCha1&ct=&position=JH129039%3A189769-189769) [D](https://genome.ucsc.edu/cgi-bin/hgc?o=189768&g=getDna&i=JH129039&c=JH129039&l=189768&r=189769&db=latCha1) Coelacanth c  [B](https://genome.ucsc.edu/cgi-bin/hgTracks?db=tetNig2&ct=&position=chr1%3A1170603-1170603) [D](https://genome.ucsc.edu/cgi-bin/hgc?o=1170602&g=getDna&i=chr1&c=chr1&l=1170602&r=1170603&db=tetNig2) Tetraodon c  [B](https://genome.ucsc.edu/cgi-bin/hgTracks?db=fr3&ct=&position=chr8%3A8424484-8424484) [D](https://genome.ucsc.edu/cgi-bin/hgc?o=8424483&g=getDna&i=chr8&c=chr8&l=8424483&r=8424484&db=fr3) Fugu c  Yellowbelly pufferfish c  [B](https://genome.ucsc.edu/cgi-bin/hgTracks?db=oreNil2&ct=&position=chrLG2%3A20189091-20189091) [D](https://genome.ucsc.edu/cgi-bin/hgc?o=20189090&g=getDna&i=chrLG2&c=chrLG2&l=20189090&r=20189091&db=oreNil2) Nile tilapia c  Princess of Burundi c  Burton's mouthbreeder c  Zebra mbuna c  Pundamilia nyererei c  [B](https://genome.ucsc.edu/cgi-bin/hgTracks?db=oryLat2&ct=&position=chr10%3A13461129-13461129) [D](https://genome.ucsc.edu/cgi-bin/hgc?o=13461128&g=getDna&i=chr10&c=chr10&l=13461128&r=13461129&db=oryLat2) Medaka c  Southern platyfish c  [B](https://genome.ucsc.edu/cgi-bin/hgTracks?db=gasAcu1&ct=&position=chrIV%3A11757060-11757060) [D](https://genome.ucsc.edu/cgi-bin/hgc?o=11757059&g=getDna&i=chrIV&c=chrIV&l=11757059&r=11757060&db=gasAcu1) Stickleback c  [B](https://genome.ucsc.edu/cgi-bin/hgTracks?db=gadMor1&ct=&position=HE567515%3A36300-36300) [D](https://genome.ucsc.edu/cgi-bin/hgc?o=36299&g=getDna&i=HE567515&c=HE567515&l=36299&r=36300&db=gadMor1&hgSeq.revComp=on) Atlantic cod c  [B](https://genome.ucsc.edu/cgi-bin/hgTracks?db=danRer7&ct=&position=chr6%3A10040124-10040124) [D](https://genome.ucsc.edu/cgi-bin/hgc?o=10040123&g=getDna&i=chr6&c=chr6&l=10040123&r=10040124&db=danRer7&hgSeq.revComp=on) Zebrafish c  Mexican tetra (cavefish) c  Spotted gar c  [B](https://genome.ucsc.edu/cgi-bin/hgTracks?db=petMar2&ct=&position=GL480158%3A14820-14820) [D](https://genome.ucsc.edu/cgi-bin/hgc?o=14819&g=getDna&i=GL480158&c=GL480158&l=14819&r=14820&db=petMar2&hgSeq.revComp=on) Lamprey c  [B](https://genome.ucsc.edu/cgi-bin/hgTracks?db=susScr3&ct=&position=chr11%3A16222894-16243579) [D](https://genome.ucsc.edu/cgi-bin/hgc?o=16222893&g=getDna&i=chr11&c=chr11&l=16222893&r=16243579&db=susScr3&hgSeq.revComp=on) Pig -  [B](https://genome.ucsc.edu/cgi-bin/hgTracks?db=ornAna1&ct=&position=chr6%3A11364587-11364811) [D](https://genome.ucsc.edu/cgi-bin/hgc?o=11364586&g=getDna&i=chr6&c=chr6&l=11364586&r=11364811&db=ornAna1&hgSeq.revComp=on) Platypus =  [B](https://genome.ucsc.edu/cgi-bin/hgTracks?db=macEug2&ct=&position=GL110657%3A9333-12885) [D](https://genome.ucsc.edu/cgi-bin/hgc?o=9332&g=getDna&i=GL110657&c=GL110657&l=9332&r=12885&db=macEug2&hgSeq.revComp=on) Wallaby =  [Data schema/format description and download](https://genome.ucsc.edu/cgi-bin/hgTables?db=hg19&hgta_group=compGeno&hgta_track=cons100way&hgta_table=multiz100way&hgta_doSchema=describe+table+schema)  [Go to Conservation track controls](https://genome.ucsc.edu/cgi-bin/hgTrackUi?g=cons100way&hgsid=2306903998_81N7R1Zas6CL9LKWAfmjniqz8ti7)  **Data last updated at UCSC:**2014-02-07  Downloads for data in this track are available:   - [Multiz alignments](http://hgdownload.soe.ucsc.edu/goldenPath/hg19/multiz100way/) (MAF format), and phylogenetic trees - [PhyloP conservation](http://hgdownload.soe.ucsc.edu/goldenPath/hg19/phyloP100way/) (WIG format) - [PhastCons conservation](http://hgdownload.soe.ucsc.edu/goldenPath/hg19/phastCons100way/) (WIG format)   **Description**  This track shows multiple alignments of 100 vertebrate species and measurements of evolutionary conservation using two methods (*phastCons* and *phyloP*) from the [PHAST package](http://compgen.cshl.edu/phast/#_blank), for all species. The multiple alignments were generated using multiz and other tools in the UCSC/[Penn State Bioinformatics](http://www.bx.psu.edu/miller_lab/) comparative genomics alignment pipeline. Conserved elements identified by phastCons are also displayed in this track. PHAST/Multiz are built from chains ("alignable") and nets ("syntenic"), see the documentation of the Chain/Net tracks for a description of the complete alignment process.  PhastCons is a hidden Markov model-based method that estimates the probability that each nucleotide belongs to a conserved element, based on the multiple alignment. It considers not just each individual alignment column, but also its flanking columns. By contrast, phyloP separately measures conservation at individual columns, ignoring the effects of their neighbors. As a consequence, the phyloP plots have a less smooth appearance than the phastCons plots, with more "texture" at individual sites. The two methods have different strengths and weaknesses. PhastCons is sensitive to "runs" of conserved sites, and is therefore effective for picking out conserved elements. PhyloP, on the other hand, is more appropriate for evaluating signatures of selection at particular nucleotides or classes of nucleotides (e.g., third codon positions, or first positions of miRNA target sites).  Another important difference is that phyloP can measure acceleration (faster evolution than expected under neutral drift) as well as conservation (slower than expected evolution). In the phyloP plots, sites predicted to be conserved are assigned positive scores (and shown in blue), while sites predicted to be fast-evolving are assigned negative scores (and shown in red). The absolute values of the scores represent -log p-values under a null hypothesis of neutral evolution. The phastCons scores, by contrast, represent probabilities of negative selection and range between 0 and 1.  Both phastCons and phyloP treat alignment gaps and unaligned nucleotides as missing data, and both were run with the same parameters.  UCSC has repeatmasked and aligned all genome assemblies, and provides all the sequences for download. For genome assemblies not available in the genome browser, there are alternative assembly hub genome browsers. Missing sequence in any assembly is highlighted in the track display by regions of yellow when zoomed out and by Ns when displayed at base level (see *Gap Annotation*, below).   \| **Primate subset** \| \| \| \| \| \| --- \| --- \| --- \| --- \| --- \| \| **Organism** \| **Species** \| **Release date** \| **UCSC version** \| **Alignment type** \| \| Baboon \| Papio hamadryas \| Nov 2008 \| [Baylor Pham_1.0/papHam1](https://genome.ucsc.edu/cgi-bin/hgGateway?db=papHam1) \| Reciprocal best net \| \| Bushbaby \| Otolemur garnettii \| Mar 2011 \| [Broad/otoGar3](https://genome.ucsc.edu/cgi-bin/hgGateway?db=otoGar3) \| Syntenic net \| \| Chimp \| Pan troglodytes \| Feb 2011 \| [CSAC 2.1.4/panTro4](https://genome.ucsc.edu/cgi-bin/hgGateway?db=panTro4) \| Syntenic net \| \| Crab-eating macaque \| Macaca fascicularis \| Jun 2013 \| [Macaca_fascicularis_5.0/macFas5](ftp://hgdownload.soe.ucsc.edu/gbdb/macFas5) \| Syntenic net \| \| Gibbon \| Nomascus leucogenys \| Oct 2012 \| [GGSC Nleu3.0/nomLeu3](https://genome.ucsc.edu/cgi-bin/hgGateway?db=nomLeu3) \| Syntenic net \| \| Gorilla \| Gorilla gorilla gorilla \| May 2011 \| [gorGor3.1/gorGor3](https://genome.ucsc.edu/cgi-bin/hgGateway?db=gorGor3) \| Reciprocal best net \| \| Green monkey \| Chlorocebus sabaeus \| Jun 2013 \| [Chlorocebus_sabeus 1.0/chlSab1](ftp://hgdownload.soe.ucsc.edu/gbdb/chlSab1) \| Syntenic net \| \| Human \| Homo sapiens \| Feb 2009 \| [GRCh37/hg19](https://genome.ucsc.edu/cgi-bin/hgGateway?db=hg19) \| reference species \| \| Marmoset \| Callithrix jacchus \| Mar 2009 \| [WUGSC 3.2/calJac3](https://genome.ucsc.edu/cgi-bin/hgGateway?db=calJac3) \| Syntenic net \| \| Orangutan \| Pongo pygmaeus abelii \| July 2007 \| [WUGSC 2.0.2/ponAbe2](https://genome.ucsc.edu/cgi-bin/hgGateway?db=ponAbe2) \| Reciprocal best net \| \| Rhesus \| Macaca mulatta \| Oct 2010 \| [BGI CR_1.0/rheMac3](https://genome.ucsc.edu/cgi-bin/hgGateway?db=rheMac3) \| Syntenic net \| \| Squirrel monkey \| Saimiri boliviensis \| Oct 2011 \| [Broad/saiBol1](https://genome.ucsc.edu/cgi-bin/hgGateway?db=saiBol1) \| Syntenic net \| \| **Euarchontoglires subset** \| \| \| \| \| \| Brush-tailed rat \| Octodon degus \| Apr 2012 \| [OctDeg1.0/octDeg1](ftp://hgdownload.soe.ucsc.edu/gbdb/octDeg1) \| Syntenic net \| \| Chinchilla \| Chinchilla lanigera \| May 2012 \| [ChiLan1.0/chiLan1](ftp://hgdownload.soe.ucsc.edu/gbdb/chiLan1) \| Syntenic net \| \| Chinese hamster \| Cricetulus griseus \| Jul 2013 \| [C_griseus_v1.0/criGri1](ftp://hgdownload.soe.ucsc.edu/gbdb/criGri1) \| Syntenic net \| \| Chinese tree shrew \| Tupaia chinensis \| Jan 2013 \| [TupChi_1.0/tupChi1](ftp://hgdownload.soe.ucsc.edu/gbdb/tupChi1) \| Syntenic net \| \| Golden hamster \| Mesocricetus auratus \| Mar 2013 \| [MesAur1.0/mesAur1](ftp://hgdownload.soe.ucsc.edu/gbdb/mesAur1) \| Syntenic net \| \| Guinea pig \| Cavia porcellus \| Feb 2008 \| [Broad/cavPor3](https://genome.ucsc.edu/cgi-bin/hgGateway?db=cavPor3) \| Syntenic net \| \| Lesser Egyptian jerboa \| Jaculus jaculus \| May 2012 \| [JacJac1.0/jacJac1](ftp://hgdownload.soe.ucsc.edu/gbdb/jacJac1) \| Syntenic net \| \| Mouse \| Mus musculus \| Dec 2011 \| [GRCm38/mm10](https://genome.ucsc.edu/cgi-bin/hgGateway?db=mm10) \| Syntenic net \| \| Naked mole-rat \| Heterocephalus glaber \| Jan 2012 \| [Broad HetGla_female_1.0/hetGla2](https://genome.ucsc.edu/cgi-bin/hgGateway?db=hetGla2) \| Syntenic net \| \| Pika \| Ochotona princeps \| May 2012 \| [OchPri3.0/ochPri3](ftp://hgdownload.soe.ucsc.edu/gbdb/ochPri3) \| Syntenic net \| \| Prairie vole \| Microtus ochrogaster \| Oct 2012 \| [MicOch1.0/micOch1](ftp://hgdownload.soe.ucsc.edu/gbdb/micOch1) \| Syntenic net \| \| Rabbit \| Oryctolagus cuniculus \| Apr 2009 \| [Broad/oryCun2](https://genome.ucsc.edu/cgi-bin/hgGateway?db=oryCun2) \| Syntenic net \| \| Rat \| Rattus norvegicus \| Mar 2012 \| [RGSC 5.0/rn5](https://genome.ucsc.edu/cgi-bin/hgGateway?db=rn5) \| Syntenic net \| \| Squirrel \| Spermophilus tridecemlineatus \| Nov 2011 \| [Broad/speTri2](https://genome.ucsc.edu/cgi-bin/hgGateway?db=speTri2) \| Syntenic net \| \| **Laurasiatheria subset** \| \| \| \| \| \| Alpaca \| Vicugna pacos \| Mar 2013 \| [Vicugna_pacos-2.0.1/vicPac2](https://genome.ucsc.edu/cgi-bin/hgGateway?db=vicPac2) \| Syntenic net \| \| Bactrian camel \| Camelus ferus \| Dec 2011 \| [CB1/camFer1](ftp://hgdownload.soe.ucsc.edu/gbdb/camFer1) \| Syntenic net \| \| Big brown bat \| Eptesicus fuscus \| Jul 2012 \| [EptFus1.0/eptFus1](ftp://hgdownload.soe.ucsc.edu/gbdb/eptFus1) \| Syntenic net \| \| Black flying-fox \| Pteropus alecto \| Aug 2012 \| [ASM32557v1/pteAle1](ftp://hgdownload.soe.ucsc.edu/gbdb/pteAle1) \| Syntenic net \| \| Cat \| Felis catus \| Sep 2011 \| [ICGSC Felis_catus 6.2/felCat5](https://genome.ucsc.edu/cgi-bin/hgGateway?db=felCat5) \| Syntenic net \| \| Cow \| Bos taurus \| Oct 2011 \| [Baylor Btau_4.6.1/bosTau7](https://genome.ucsc.edu/cgi-bin/hgGateway?db=bosTau7) \| Syntenic net \| \| David's myotis bat \| Myotis davidii \| Aug 2012 \| [ASM32734v1/myoDav1](ftp://hgdownload.soe.ucsc.edu/gbdb/myoDav1) \| Syntenic net \| \| Dog \| Canis lupus familiaris \| Sep 2011 \| [Broad CanFam3.1/canFam3](https://genome.ucsc.edu/cgi-bin/hgGateway?db=canFam3) \| Syntenic net \| \| Dolphin \| Tursiops truncatus \| Oct 2011 \| [Baylor Ttru_1.4/turTru2](https://genome.ucsc.edu/cgi-bin/hgGateway?db=turTru2) \| Reciprocal best net \| \| Domestic goat \| Capra hircus \| May 2012 \| [CHIR_1.0/capHir1](ftp://hgdownload.soe.ucsc.edu/gbdb/capHir1) \| Syntenic net \| \| Ferret \| Mustela putorius furo \| Apr 2011 \| [MusPutFur1.0/musFur1](https://genome.ucsc.edu/cgi-bin/hgGateway?db=musFur1) \| Syntenic net \| \| Hedgehog \| Erinaceus europaeus \| May 2012 \| [EriEur2.0/eriEur2](ftp://hgdownload.soe.ucsc.edu/gbdb/eriEur2) \| Syntenic net \| \| Horse \| Equus caballus \| Sep 2007 \| [Broad/equCab2](https://genome.ucsc.edu/cgi-bin/hgGateway?db=equCab2) \| Syntenic net \| \| Killer whale \| Orcinus orca \| Jan 2013 \| [Oorc_1.1/orcOrc1](ftp://hgdownload.soe.ucsc.edu/gbdb/orcOrc1) \| Syntenic net \| \| Little brown bat \| Myotis lucifugus \| Jul 2010 \| [Broad Institute Myoluc2.0/myoLuc2](https://genome.ucsc.edu/cgi-bin/hgGateway?db=myoLuc2) \| Syntenic net \| \| Megabat \| Pteropus vampyrus \| Jul 2008 \| [Broad/pteVam1](https://genome.ucsc.edu/cgi-bin/hgGateway?db=pteVam1) \| Reciprocal best net \| \| Pacific walrus \| Odobenus rosmarus divergens \| Jan 2013 \| [Oros_1.0/odoRosDiv1](ftp://hgdownload.soe.ucsc.edu/gbdb/odoRosDiv1) \| Syntenic net \| \| Panda \| Ailuropoda melanoleuca \| Dec 2009 \| [BGI-Shenzhen 1.0/ailMel1](https://genome.ucsc.edu/cgi-bin/hgGateway?db=ailMel1) \| Syntenic net \| \| Pig \| Sus scrofa \| Aug 2011 \| [SGSC Sscrofa10.2/susScr3](https://genome.ucsc.edu/cgi-bin/hgGateway?db=susScr3) \| Syntenic net \| \| Sheep \| Ovis aries \| Aug 2012 \| [ISGC Oar_v3.1/oviAri3](https://genome.ucsc.edu/cgi-bin/hgGateway?db=oviAri3) \| Syntenic net \| \| Shrew \| Sorex araneus \| Aug 2008 \| [Broad/sorAra2](ftp://hgdownload.soe.ucsc.edu/gbdb/sorAra2) \| Syntenic net \| \| Star-nosed mole \| Condylura cristata \| Mar 2012 \| [ConCri1.0/conCri1](ftp://hgdownload.soe.ucsc.edu/gbdb/conCri1) \| Syntenic net \| \| Tibetan antelope \| Pantholops hodgsonii \| May 2013 \| [PHO1.0/panHod1](ftp://hgdownload.soe.ucsc.edu/gbdb/panHod1) \| Syntenic net \| \| Weddell seal \| Leptonychotes weddellii \| Mar 2013 \| [LepWed1.0/lepWed1](ftp://hgdownload.soe.ucsc.edu/gbdb/lepWed1) \| Reciprocal best net \| \| White rhinoceros \| Ceratotherium simum \| May 2012 \| [CerSimSim1.0/cerSim1](https://genome.ucsc.edu/cgi-bin/hgGateway?db=cerSim1) \| Syntenic net \| \| **Afrotheria subset** \| \| \| \| \| \| Aardvark \| Orycteropus afer afer \| May 2012 \| [OryAfe1.0/oryAfe1](ftp://hgdownload.soe.ucsc.edu/gbdb/oryAfe1) \| Syntenic net \| \| Cape elephant shrew \| Elephantulus edwardii \| Aug 2012 \| [EleEdw1.0/eleEdw1](ftp://hgdownload.soe.ucsc.edu/gbdb/eleEdw1) \| Syntenic net \| \| Cape golden mole \| Chrysochloris asiatica \| Aug 2012 \| [ChrAsi1.0/chrAsi1](ftp://hgdownload.soe.ucsc.edu/gbdb/chrAsi1) \| Syntenic net \| \| Elephant \| Loxodonta africana \| Jul 2009 \| [Broad/loxAfr3](https://genome.ucsc.edu/cgi-bin/hgGateway?db=loxAfr3) \| Syntenic net \| \| Manatee \| Trichechus manatus latirostris \| Oct 2011 \| [Broad v1.0/triMan1](https://genome.ucsc.edu/cgi-bin/hgGateway?db=triMan1) \| Syntenic net \| \| Tenrec \| Echinops telfairi \| Nov 2012 \| [Broad/echTel2](https://genome.ucsc.edu/cgi-bin/hgGateway?db=echTel2) \| Syntenic net \| \| **Mammal subset** \| \| \| \| \| \| Armadillo \| Dasypus novemcinctus \| Dec 2011 \| [Baylor/dasNov3](https://genome.ucsc.edu/cgi-bin/hgGateway?db=dasNov3) \| Syntenic net \| \| Opossum \| Monodelphis domestica \| Oct 2006 \| [Broad/monDom5](https://genome.ucsc.edu/cgi-bin/hgGateway?db=monDom5) \| Net \| \| Platypus \| Ornithorhynchus anatinus \| Mar 2007 \| [WUGSC 5.0.1/ornAna1](https://genome.ucsc.edu/cgi-bin/hgGateway?db=ornAna1) \| Reciprocal best net \| \| Tasmanian devil \| Sarcophilus harrisii \| Feb 2011 \| [WTSI Devil_ref v7.0/sarHar1](https://genome.ucsc.edu/cgi-bin/hgGateway?db=sarHar1) \| Net \| \| Wallaby \| Macropus eugenii \| Sep 2009 \| [TWGS Meug_1.1/macEug2](https://genome.ucsc.edu/cgi-bin/hgGateway?db=macEug2) \| Reciprocal best net \| \| **Aves subset** \| \| \| \| \| \| Budgerigar \| Melopsittacus undulatus \| Sep 2011 \| [WUSTL v6.3/melUnd1](https://genome.ucsc.edu/cgi-bin/hgGateway?db=melUnd1) \| Net \| \| Chicken \| Gallus gallus \| Nov 2011 \| [ICGSC Gallus_gallus-4.0/galGal4](https://genome.ucsc.edu/cgi-bin/hgGateway?db=galGal4) \| Net \| \| Collared flycatcher \| Ficedula albicollis \| Jun 2013 \| [FicAlb1.5/ficAlb2](ftp://hgdownload.soe.ucsc.edu/gbdb/ficAlb2) \| Net \| \| Mallard duck \| Anas platyrhynchos \| Apr 2013 \| [BGI_duck_1.0/anaPla1](ftp://hgdownload.soe.ucsc.edu/gbdb/anaPla1) \| Net \| \| Medium ground finch \| Geospiza fortis \| Apr 2012 \| [GeoFor_1.0/geoFor1](https://genome.ucsc.edu/cgi-bin/hgGateway?db=geoFor1) \| Net \| \| Parrot \| Amazona vittata \| Jan 2013 \| [AV1/amaVit1](ftp://hgdownload.soe.ucsc.edu/gbdb/amaVit1) \| Net \| \| Peregrine falcon \| Falco peregrinus \| Feb 2013 \| [F_peregrinus_v1.0/falPer1](ftp://hgdownload.soe.ucsc.edu/gbdb/falPer1) \| Net \| \| Rock pigeon \| Columba livia \| Feb 2013 \| [Cliv_1.0/colLiv1](ftp://hgdownload.soe.ucsc.edu/gbdb/colLiv1) \| Net \| \| Saker falcon \| Falco cherrug \| Feb 2013 \| [F_cherrug_v1.0/falChe1](ftp://hgdownload.soe.ucsc.edu/gbdb/falChe1) \| Net \| \| Scarlet macaw \| Ara macao \| Jun 2013 \| [SMACv1.1/araMac1](ftp://hgdownload.soe.ucsc.edu/gbdb/araMac1) \| Net \| \| Tibetan ground jay \| Pseudopodoces humilis \| Jan 2013 \| [PseHum1.0/pseHum1](ftp://hgdownload.soe.ucsc.edu/gbdb/pseHum1) \| Net \| \| Turkey \| Meleagris gallopavo \| Dec 2009 \| [TGC Turkey_2.01/melGal1](https://genome.ucsc.edu/cgi-bin/hgGateway?db=melGal1) \| Net \| \| White-throated sparrow \| Zonotrichia albicollis \| Apr 2013 \| [ASM38545v1/zonAlb1](ftp://hgdownload.soe.ucsc.edu/gbdb/zonAlb1) \| Net \| \| Zebra finch \| Taeniopygia guttata \| Feb 2013 \| [WashU taeGut324/taeGut2](ftp://hgdownload.soe.ucsc.edu/gbdb/taeGut2) \| Net \| \| **Sarcopterygii subset** \| \| \| \| \| \| American alligator \| Alligator mississippiensis \| Aug 2012 \| [allMis0.2/allMis1](ftp://hgdownload.soe.ucsc.edu/gbdb/allMis1) \| Net \| \| Chinese softshell turtle \| Pelodiscus sinensis \| Oct 2011 \| [PelSin_1.0/pelSin1](ftp://hgdownload.soe.ucsc.edu/gbdb/pelSin1) \| Net \| \| Coelacanth \| Latimeria chalumnae \| Aug 2011 \| [Broad/latCha1](https://genome.ucsc.edu/cgi-bin/hgGateway?db=latCha1) \| Net \| \| Green seaturtle \| Chelonia mydas \| Mar 2013 \| [CheMyd_1.0/cheMyd1](ftp://hgdownload.soe.ucsc.edu/gbdb/cheMyd1) \| Net \| \| Lizard \| Anolis carolinensis \| May 2010 \| [Broad AnoCar2.0/anoCar2](https://genome.ucsc.edu/cgi-bin/hgGateway?db=anoCar2) \| Net \| \| Painted turtle \| Chrysemys picta bellii \| Dec 2011 \| [v3.0.1/chrPic1](https://genome.ucsc.edu/cgi-bin/hgGateway?db=chrPic1) \| Net \| \| Spiny softshell turtle \| Apalone spinifera \| May 2013 \| [ASM38561v1/apaSpi1](ftp://hgdownload.soe.ucsc.edu/gbdb/apaSpi1) \| Net \| \| X. tropicalis \| Xenopus tropicalis \| Sep 2012 \| [JGI 7.0/xenTro7](ftp://hgdownload.soe.ucsc.edu/gbdb/xenTro7) \| Net \| \| **Fish subset** \| \| \| \| \| \| Atlantic cod \| Gadus morhua \| May 2010 \| [Genofisk GadMor_May2010/gadMor1](https://genome.ucsc.edu/cgi-bin/hgGateway?db=gadMor1) \| Net \| \| Burton's mouthbreeder \| Haplochromis burtoni \| Oct 2011 \| [AstBur1.0/hapBur1](ftp://hgdownload.soe.ucsc.edu/gbdb/hapBur1) \| Net \| \| Fugu \| Takifugu rubripes \| Oct 2011 \| [FUGU5/fr3](ftp://hgdownload.soe.ucsc.edu/gbdb/fr3) \| Net \| \| Lamprey \| Petromyzon marinus \| Sep 2010 \| [WUGSC 7.0/petMar2](https://genome.ucsc.edu/cgi-bin/hgGateway?db=petMar2) \| Net \| \| Medaka \| Oryzias latipes \| Oct 2005 \| [NIG/UT MEDAKA1/oryLat2](https://genome.ucsc.edu/cgi-bin/hgGateway?db=oryLat2) \| Net \| \| Mexican tetra (cavefish) \| Astyanax mexicanus \| Apr 2013 \| [Astyanax_mexicanus-1.0.2/astMex1](ftp://hgdownload.soe.ucsc.edu/gbdb/astMex1) \| Net \| \| Nile tilapia \| Oreochromis niloticus \| Jan 2011 \| [Broad oreNil1.1/oreNil2](https://genome.ucsc.edu/cgi-bin/hgGateway?db=oreNil2) \| Net \| \| Princess of Burundi \| Neolamprologus brichardi \| May 2011 \| [NeoBri1.0/neoBri1](ftp://hgdownload.soe.ucsc.edu/gbdb/neoBri1) \| Net \| \| Pundamilia nyererei \| Pundamilia nyererei \| Oct 2011 \| [PunNye1.0/punNye1](ftp://hgdownload.soe.ucsc.edu/gbdb/punNye1) \| Net \| \| Southern platyfish \| Xiphophorus maculatus \| Jan 2012 \| [Xiphophorus_maculatus-4.4.2/xipMac1](ftp://hgdownload.soe.ucsc.edu/gbdb/xipMac1) \| Net \| \| Spotted gar \| Lepisosteus oculatus \| Dec 2011 \| [LepOcu1/lepOcu1](ftp://hgdownload.soe.ucsc.edu/gbdb/lepOcu1) \| Net \| \| Stickleback \| Gasterosteus aculeatus \| Feb 2006 \| [Broad/gasAcu1](https://genome.ucsc.edu/cgi-bin/hgGateway?db=gasAcu1) \| Net \| \| Tetraodon \| Tetraodon nigroviridis \| Mar 2007 \| [Genoscope 8.0/tetNig2](https://genome.ucsc.edu/cgi-bin/hgGateway?db=tetNig2) \| Net \| \| Yellowbelly pufferfish \| Takifugu flavidus \| May 2013 \| [version 1 of Takifugu flavidus genome/takFla1](ftp://hgdownload.soe.ucsc.edu/gbdb/takFla1) \| Net \| \| Zebra mbuna \| Maylandia zebra \| Mar 2012 \| [MetZeb1.1/mayZeb1](ftp://hgdownload.soe.ucsc.edu/gbdb/mayZeb1) \| Net \| \| Zebrafish \| Danio rerio \| Jul 2010 \| [Zv9/danRer7](https://genome.ucsc.edu/cgi-bin/hgGateway?db=danRer7) \| Net \|   **Table 1.** *Genome assemblies included in the 100-way Conservation track.*  **Display Conventions and Configuration**  In full and pack display modes, conservation scores are displayed as a *wiggle track* (histogram) in which the height reflects the size of the score. The conservation wiggles can be configured in a variety of ways to highlight different aspects of the displayed information. Click the [Graph configuration help](https://genome.ucsc.edu/goldenPath/help/hgWiggleTrackHelp.html) link for an explanation of the configuration options.  Pairwise alignments of each species to the human genome are displayed below the conservation histogram as a grayscale density plot (in pack mode) or as a wiggle (in full mode) that indicates alignment quality. In dense display mode, conservation is shown in grayscale using darker values to indicate higher levels of overall conservation as scored by phastCons.  Checkboxes on the track configuration page allow selection of the species to include in the pairwise display. Note that excluding species from the pairwise display does not alter the the conservation score display.  To view detailed information about the alignments at a specific position, zoom the display in to 30,000 or fewer bases, then click on the alignment.  **Gap Annotation**  The *Display chains between alignments* configuration option enables display of gaps between alignment blocks in the pairwise alignments in a manner similar to the Chain track display. The following conventions are used:   - **Single line:** No bases in the aligned species. Possibly due to a lineage-specific insertion between the aligned blocks in the human genome or a lineage-specific deletion between the aligned blocks in the aligning species. - **Double line:** Aligning species has one or more unalignable bases in the gap region. Possibly due to excessive evolutionary distance between species or independent indels in the region between the aligned blocks in both species. - **Pale yellow coloring:** Aligning species has Ns in the gap region. Reflects uncertainty in the relationship between the DNA of both species, due to lack of sequence in relevant portions of the aligning species.   **Genomic Breaks**  Discontinuities in the genomic context (chromosome, scaffold or region) of the aligned DNA in the aligning species are shown as follows:   - **Vertical blue bar:** Represents a discontinuity that persists indefinitely on either side, *e.g.* a large region of DNA on either side of the bar comes from a different chromosome in the aligned species due to a large scale rearrangement. - **Green square brackets:** Enclose shorter alignments consisting of DNA from one genomic context in the aligned species nested inside a larger chain of alignments from a different genomic context. The alignment within the brackets may represent a short misalignment, a lineage-specific insertion of a transposon in the human genome that aligns to a paralogous copy somewhere else in the aligned species, or other similar occurrence.   **Base Level**  When zoomed-in to the base-level display, the track shows the base composition of each alignment. The numbers and symbols on the Gaps line indicate the lengths of gaps in the human sequence at those alignment positions relative to the longest non-human sequence. If there is sufficient space in the display, the size of the gap is shown. If the space is insufficient and the gap size is a multiple of 3, a "*" is displayed; other gap sizes are indicated by "+".  Codon translation is available in base-level display mode if the displayed region is identified as a coding segment. To display this annotation, select the species for translation from the pull-down menu in the Codon Translation configuration section at the top of the page. Then, select one of the following modes:   - **No codon translation:** The gene annotation is not used; the bases are displayed without translation. - **Use default species reading frames for translation:** The annotations from the genome displayed in the *Default species to establish reading frame* pull-down menu are used to translate all the aligned species present in the alignment. - **Use reading frames for species if available, otherwise no translation:** Codon translation is performed only for those species where the region is annotated as protein coding. - **Use reading frames for species if available, otherwise use default species:** Codon translation is done on those species that are annotated as being protein coding over the aligned region using species-specific annotation; the remaining species are translated using the default species annotation.   Codon translation uses the following gene tracks as the basis for translation:   \| **Gene Track** \| **Species** \| \| --- \| --- \| \| UCSC Genes \| Human, Mouse \| \| RefSeq Genes \| Cow, Frog (X. tropicalis) \| \| Ensembl Genes v73 \| Atlantic cod, Bushbaby, Cat, Chicken, Chimp, Coelacanth, Dog, Elephant, Ferret, Fugu, Gorilla, Horse, Lamprey, Little brown bat, Lizard, Mallard duck, Marmoset, Medaka, Orangutan, Panda, Pig, Platypus, Rat, Soft-shell Turtle, Southern platyfish, Squirrel, Tasmanian devil, Tetraodon, Zebrafish \| \| no annotation \| Aardvark, Alpaca, American alligator, Armadillo, Baboon, Bactrian camel, Big brown bat, Black flying-fox, Brush-tailed rat, Budgerigar, Burton's mouthbreeder, Cape elephant shrew, Cape golden mole, Chinchilla, Chinese hamster, Chinese tree shrew, Collared flycatcher, Crab-eating macaque, David's myotis (bat), Dolphin, Domestic goat, Gibbon, Golden hamster, Green monkey, Green seaturtle, Hedgehog, Killer whale, Lesser Egyptian jerboa, Manatee, Medium ground finch, Mexican tetra (cavefish), Naked mole-rat, Nile tilapia, Pacific walrus, Painted turtle, Parrot, Peregrine falcon, Pika, Prairie vole, Princess of Burundi, Pundamilia nyererei, Rhesus, Rock pigeon, Saker falcon, Scarlet Macaw, Sheep, Shrew, Spiny softshell turtle, Spotted gar, Squirrel monkey, Star-nosed mole, Tawny puffer fish, Tenrec, Tibetan antelope, Tibetan ground jay, Wallaby, Weddell seal, White rhinoceros, White-throated sparrow, Zebra Mbuna, Zebra finch \|   **Table 2.** *Gene tracks used for codon translation.*  **Methods**  Pairwise alignments with the human genome were generated for each species using lastz from repeat-masked genomic sequence. Pairwise alignments were then linked into chains using a dynamic programming algorithm that finds maximally scoring chains of gapless subsections of the alignments organized in a kd-tree. The scoring matrix and parameters for pairwise alignment and chaining were tuned for each species based on phylogenetic distance from the reference. High-scoring chains were then placed along the genome, with gaps filled by lower-scoring chains, to produce an alignment net. For more information about the chaining and netting process and parameters for each species, see the description pages for the Chain and Net tracks.  An additional filtering step was introduced in the generation of the 60-way conservation track to reduce the number of paralogs and pseudogenes from the high-quality assemblies and the suspect alignments from the low-quality assemblies: the pairwise alignments of high-quality mammalian sequences (placental and marsupial) were filtered based on synteny; those for 2X mammalian genomes were filtered to retain only alignments of best quality in both the target and query ("reciprocal best").  The resulting best-in-genome pairwise alignments were progressively aligned using multiz/autoMZ, following the tree topology diagrammed above, to produce multiple alignments. The multiple alignments were post-processed to add annotations indicating alignment gaps, genomic breaks, and base quality of the component sequences. The annotated multiple alignments, in MAF format, are available for [bulk download](http://hgdownload.soe.ucsc.edu/goldenPath/hg19/multiz100way). An alignment summary table containing an entry for each alignment block in each species was generated to improve track display performance at large scales. Framing tables were constructed to enable visualization of codons in the multiple alignment display.  **Phylogenetic Tree Model**  Both *phastCons* and *phyloP* are phylogenetic methods that rely on a tree model containing the tree topology, branch lengths representing evolutionary distance at neutrally evolving sites, the background distribution of nucleotides, and a substitution rate matrix. The [all-species tree model](http://hgdownload.soe.ucsc.edu/goldenPath/hg19/phastCons100way/hg19.100way.phastCons.mod) for this track was generated using the *phyloFit* program from the PHAST package (REV model, EM algorithm, medium precision) using multiple alignments of 4-fold degenerate sites extracted from the 60-way alignment (msa_view). The 4d sites were derived from the RefSeq (Reviewed+Coding) gene set, filtered to select single-coverage long transcripts.  This same tree model was used in the phyloP calculations; however, the background frequencies were modified to maintain reversibility. The resulting tree model: [all species](http://hgdownload.soe.ucsc.edu/goldenPath/hg19/phyloP100way/hg19.100way.phyloP100way.mod).  **PhastCons Conservation**  The phastCons program computes conservation scores based on a phylo-HMM, a type of probabilistic model that describes both the process of DNA substitution at each site in a genome and the way this process changes from one site to the next (Felsenstein and Churchill 1996, Yang 1995, Siepel and Haussler 2005). PhastCons uses a two-state phylo-HMM, with a state for conserved regions and a state for non-conserved regions. The value plotted at each site is the posterior probability that the corresponding alignment column was "generated" by the conserved state of the phylo-HMM. These scores reflect the phylogeny (including branch lengths) of the species in question, a continuous-time Markov model of the nucleotide substitution process, and a tendency for conservation levels to be autocorrelated along the genome (i.e., to be similar at adjacent sites). The general reversible (REV) substitution model was used. Unlike many conservation-scoring programs, phastCons does not rely on a sliding window of fixed size; therefore, short highly-conserved regions and long moderately conserved regions can both obtain high scores. More information about phastCons can be found in Siepel *et al*. 2005.  The phastCons parameters used were: expected-length=45, target-coverage=0.3, rho=0.3.  **PhyloP Conservation**  The phyloP program supports several different methods for computing p-values of conservation or acceleration, for individual nucleotides or larger elements (<http://compgen.cshl.edu/phast/>). Here it was used to produce separate scores at each base (--wig-scores option), considering all branches of the phylogeny rather than a particular subtree or lineage (i.e., the --subtree option was not used). The scores were computed by performing a likelihood ratio test at each alignment column (--method LRT), and scores for both conservation and acceleration were produced (--mode CONACC).  **Conserved Elements**  The conserved elements were predicted by running phastCons with the --viterbi option. The predicted elements are segments of the alignment that are likely to have been "generated" by the conserved state of the phylo-HMM. Each element is assigned a log-odds score equal to its log probability under the conserved model minus its log probability under the non-conserved model. The "score" field associated with this track contains transformed log-odds scores, taking values between 0 and 1000. (The scores are transformed using a monotonic function of the form a * log(x) + b.) The raw log odds scores are retained in the "name" field and can be seen on the details page or in the browser when the track's display mode is set to "pack" or "full".  **Credits**  This track was created using the following programs:   - Alignment tools: lastz (formerly blastz) and multiz by Minmei Hou, Scott Schwartz and Webb Miller of the [Penn State Bioinformatics Group](http://www.bx.psu.edu/miller_lab/) - Chaining and Netting: axtChain, chainNet by Jim Kent at UCSC - Conservation scoring: phastCons, phyloP, phyloFit, tree_doctor, msa_view and other programs in PHAST by [Adam Siepel](http://siepellab.labsites.cshl.edu/) at Cold Spring Harbor Laboratory (original development done at the Haussler lab at UCSC). - MAF Annotation tools: mafAddIRows by Brian Raney, UCSC; mafAddQRows by Richard Burhans, Penn State; genePredToMafFrames by Mark Diekhans, UCSC - Tree image generator: phyloPng by Galt Barber, UCSC - Conservation track display: Kate Rosenbloom, Hiram Clawson (wiggle display), and Brian Raney (gap annotation and codon framing) at UCSC   The phylogenetic tree is based on Murphy *et al*. (2001) and general consensus in the vertebrate phylogeny community. Thanks to Giacomo Bernardi for help with the fish relationships.  **References**  **Phylo-HMMs, phastCons, and phyloP:**  Felsenstein J, Churchill GA. [A Hidden Markov Model approach to variation among sites in rate of evolution](https://academic.oup.com/mbe/article/13/1/93/1055515). *Mol Biol Evol*. 1996 Jan;13(1):93-104. PMID: [8583911](https://www.ncbi.nlm.nih.gov/pubmed/8583911)  Pollard KS, Hubisz MJ, Rosenbloom KR, Siepel A. [Detection of nonneutral substitution rates on mammalian phylogenies](https://genome.cshlp.org/content/20/1/110.long). *Genome Res*. 2010 Jan;20(1):110-21. PMID: [19858363](https://www.ncbi.nlm.nih.gov/pubmed/19858363); PMC: [PMC2798823](https://www.ncbi.nlm.nih.gov/pmc/articles/PMC2798823/)  Siepel A, Bejerano G, Pedersen JS, Hinrichs AS, Hou M, Rosenbloom K, Clawson H, Spieth J, Hillier LW, Richards S, *et al.* [Evolutionarily conserved elements in vertebrate, insect, worm, and yeast genomes](https://genome.cshlp.org/content/15/8/1034). *Genome Res*. 2005 Aug;15(8):1034-50. PMID: [16024819](https://www.ncbi.nlm.nih.gov/pubmed/16024819); PMC: [PMC1182216](https://www.ncbi.nlm.nih.gov/pmc/articles/PMC1182216/)  Siepel A, Haussler D. [Phylogenetic Hidden Markov Models](http://compgen.cshl.edu/~acs/phylohmm.pdf). In: Nielsen R, editor. Statistical Methods in Molecular Evolution. New York: Springer; 2005. pp. 325-351.  Yang Z. [A space-time process model for the evolution of DNA sequences](https://www.genetics.org/content/139/2/993). *Genetics*. 1995 Feb;139(2):993-1005. PMID: [7713447](https://www.ncbi.nlm.nih.gov/pubmed/7713447); PMC: [PMC1206396](https://www.ncbi.nlm.nih.gov/pmc/articles/PMC1206396/)  **Chain/Net:**  Kent WJ, Baertsch R, Hinrichs A, Miller W, Haussler D. [Evolution's cauldron: duplication, deletion, and rearrangement in the mouse and human genomes](https://www.pnas.org/content/100/20/11484). *Proc Natl Acad Sci U S A*. 2003 Sep 30;100(20):11484-9. PMID: [14500911](https://www.ncbi.nlm.nih.gov/pubmed/14500911); PMC: [PMC208784](https://www.ncbi.nlm.nih.gov/pmc/articles/PMC208784/)  **Multiz:**  Blanchette M, Kent WJ, Riemer C, Elnitski L, Smit AF, Roskin KM, Baertsch R, Rosenbloom K, Clawson H, Green ED, *et al.* [Aligning multiple genomic sequences with the threaded blockset aligner](https://genome.cshlp.org/content/14/4/708.abstract). *Genome Res*. 2004 Apr;14(4):708-15. PMID: [15060014](https://www.ncbi.nlm.nih.gov/pubmed/15060014); PMC: [PMC383317](https://www.ncbi.nlm.nih.gov/pmc/articles/PMC383317/)  **Lastz (formerly Blastz):**  Chiaromonte F, Yap VB, Miller W. [Scoring pairwise genomic sequence alignments](http://psb.stanford.edu/psb-online/proceedings/psb02/chiaromonte.pdf). *Pac Symp Biocomput*. 2002:115-26. PMID: [11928468](https://www.ncbi.nlm.nih.gov/pubmed/11928468)  Harris RS. [Improved pairwise alignment of genomic DNA](http://www.bx.psu.edu/~rsharris/rsharris_phd_thesis_2007.pdf). *Ph.D. Thesis*. Pennsylvania State University, USA. 2007.  Schwartz S, Kent WJ, Smit A, Zhang Z, Baertsch R, Hardison RC, Haussler D, Miller W. [Human-mouse alignments with BLASTZ](https://genome.cshlp.org/content/13/1/103.abstract). *Genome Res*. 2003 Jan;13(1):103-7. PMID: [12529312](https://www.ncbi.nlm.nih.gov/pubmed/12529312); PMC: [PMC430961](https://www.ncbi.nlm.nih.gov/pmc/articles/PMC430961/)  **Phylogenetic Tree:**  Murphy WJ, Eizirik E, O'Brien SJ, Madsen O, Scally M, Douady CJ, Teeling E, Ryder OA, Stanhope MJ, de Jong WW, Springer MS. [Resolution of the early placental mammal radiation using Bayesian phylogenetics](https://science.sciencemag.org/content/294/5550/2348). *Science*. 2001 Dec 14;294(5550):2348-51. PMID: [11743200](https://www.ncbi.nlm.nih.gov/pubmed/11743200) \|  \| \| \| --- \| --- \| --- \| --- \| --- \| --- \| --- \| --- \| --- \| --- \| --- \| --- \| --- \| --- \| --- \| --- \| --- \| --- \| --- \| --- \| --- \| --- \| --- \| --- \| --- \| --- \| --- \| --- \| --- \| --- \| --- \| --- \| --- \| --- \| --- \| --- \| --- \| --- \| --- \| --- \| --- \| --- \| --- \| --- \| --- \| --- \| --- \| --- \| --- \| --- \| --- \| --- \| --- \| --- \| --- \| --- \| --- \| --- \| --- \| --- \| --- \| --- \| --- \| --- \| --- \| --- \| --- \| --- \| --- \| --- \| --- \| --- \| --- \| --- \| --- \| --- \| --- \| --- \| --- \| --- \| --- \| --- \| --- \| --- \| --- \| --- \| --- \| --- \| --- \| --- \| --- \| --- \| --- \| --- \| --- \| --- \| --- \| --- \| --- \| --- \| --- \| --- \| --- \| --- \| --- \| --- \| --- \| --- \| --- \| --- \| --- \| --- \| --- \| --- \| --- \| --- \| --- \| --- \| --- \| --- \| --- \| --- \| --- \| --- \| --- \| --- \| --- \| --- \| --- \| --- \| --- \| --- \| --- \| --- \| --- \| --- \| --- \| --- \| --- \| --- \| --- \| --- \| --- \| --- \| --- \| --- \| --- \| --- \| --- \| --- \| --- \| --- \| --- \| --- \| --- \| --- \| --- \| --- \| --- \| --- \| --- \| --- \| --- \| --- \| --- \| --- \| --- \| --- \| --- \| --- \| --- \| --- \| --- \| --- \| --- \| --- \| --- \| --- \| --- \| --- \| --- \| --- \| --- \| --- \| --- \| --- \| --- \| --- \| --- \| --- \| --- \| --- \| --- \| --- \| --- \| --- \| --- \| --- \| --- \| --- \| --- \| --- \| --- \| --- \| --- \| --- \| --- \| --- \| --- \| --- \| --- \| --- \| --- \| --- \| --- \| --- \| --- \| --- \| --- \| --- \| --- \| --- \| --- \| --- \| --- \| --- \| --- \| --- \| --- \| --- \| --- \| --- \| --- \| --- \| --- \| --- \| --- \| --- \| --- \| --- \| --- \| --- \| --- \| --- \| --- \| --- \| --- \| --- \| --- \| --- \| --- \| --- \| --- \| --- \| --- \| --- \| --- \| --- \| --- \| --- \| --- \| --- \| --- \| --- \| --- \| --- \| --- \| --- \| --- \| --- \| --- \| --- \| --- \| --- \| --- \| --- \| --- \| --- \| --- \| --- \| --- \| --- \| --- \| --- \| --- \| --- \| --- \| --- \| --- \| --- \| --- \| --- \| --- \| --- \| --- \| --- \| --- \| --- \| --- \| --- \| --- \| --- \| --- \| --- \| --- \| --- \| --- \| --- \| --- \| --- \| --- \| --- \| --- \| --- \| --- \| --- \| --- \| --- \| --- \| --- \| --- \| --- \| --- \| --- \| --- \| --- \| --- \| --- \| --- \| --- \| --- \| --- \| --- \| --- \| --- \| --- \| --- \| --- \| --- \| --- \| --- \| --- \| --- \| --- \| --- \| --- \| --- \| --- \| --- \| --- \| --- \| --- \| --- \| --- \| --- \| --- \| --- \| --- \| --- \| --- \| --- \| --- \| --- \| --- \| --- \| --- \| --- \| --- \| --- \| --- \| --- \| --- \| --- \| --- \| --- \| --- \| --- \| --- \| --- \| --- \| --- \| --- \| --- \| --- \| --- \| --- \| --- \| --- \| --- \| --- \| --- \| --- \| --- \| --- \| --- \| --- \| --- \| --- \| --- \| --- \| --- \| --- \| --- \| --- \| --- \| --- \| --- \| --- \| --- \| --- \| --- \| --- \| --- \| --- \| --- \| --- \| --- \| --- \| --- \| --- \| --- \| --- \| --- \| --- \| --- \| --- \| --- \| --- \| --- \| --- \| --- \| --- \| --- \| --- \| --- \| --- \| --- \| --- \| --- \| --- \| --- \| --- \| --- \| --- \| --- \| --- \| --- \| --- \| --- \| --- \| --- \| --- \| --- \| --- \| --- \| --- \| --- \| --- \| --- \| --- \| --- \| --- \| --- \| --- \| --- \| --- \| --- \| --- \| --- \| --- \| --- \| --- \| --- \| --- \| --- \| --- \| --- \| --- \| --- \| --- \| --- \| --- \| --- \| --- \| --- \| --- \| --- \| --- \| --- \| --- \| --- \| --- \| --- \| --- \| --- \| --- \| --- \| --- \| --- \| --- \| --- \| --- \| --- \| --- \| --- \| --- \| --- \| --- \| --- \| --- \| --- \| --- \| --- \| --- \| --- \| --- \| --- \| --- \| --- \| --- \| --- \| --- \| --- \| --- \| --- \| --- \| --- \| --- \| --- \| --- \| --- \| --- \| --- \| --- \| --- \| --- \| --- \| --- \| --- \| --- \| --- \| --- \| --- \| --- \| --- \| --- \| --- \| --- \| --- \| --- \| --- \| --- \| --- \| --- \| --- \| --- \| --- \| --- \| --- \| --- \| \| \| --- \| --- \| --- \| --- \| --- \| --- \| --- \| --- \| --- \| --- \| --- \| --- \| --- \| --- \| --- \| --- \| --- \| --- \| --- \| --- \| --- \| --- \| --- \| --- \| --- \| --- \| --- \| --- \| --- \| --- \| --- \| --- \| --- \| --- \| --- \| --- \| --- \| --- \| --- \| --- \| --- \| --- \| --- \| --- \| --- \| --- \| --- \| --- \| --- \| --- \| --- \| --- \| --- \| --- \| --- \| --- \| --- \| --- \| --- \| --- \| --- \| --- \| --- \| --- \| --- \| --- \| --- \| --- \| --- \| --- \| --- \| --- \| --- \| --- \| --- \| --- \| --- \| --- \| --- \| --- \| --- \| --- \| --- \| --- \| --- \| --- \| --- \| --- \| --- \| --- \| --- \| --- \| --- \| --- \| --- \| --- \| --- \| --- \| --- \| --- \| --- \| --- \| --- \| --- \| --- \| --- \| --- \| --- \| --- \| --- \| --- \| --- \| --- \| --- \| --- \| --- \| --- \| --- \| --- \| --- \| --- \| --- \| --- \| --- \| --- \| --- \| --- \| --- \| --- \| --- \| --- \| --- \| --- \| --- \| --- \| --- \| --- \| --- \| --- \| --- \| --- \| --- \| --- \| --- \| --- \| --- \| --- \| --- \| --- \| --- \| --- \| --- \| --- \| --- \| --- \| --- \| --- \| --- \| --- \| --- \| --- \| --- \| --- \| --- \| --- \| --- \| --- \| --- \| --- \| --- \| --- \| --- \| --- \| --- \| --- \| --- \| --- \| --- \| --- \| --- \| --- \| --- \| --- \| --- \| --- \| --- \| --- \| --- \| --- \| --- \| --- \| --- \| --- \| --- \| --- \| --- \| --- \| --- \| --- \| --- \| --- \| --- \| --- \| --- \| --- \| --- \| --- \| --- \| --- \| --- \| --- \| --- \| --- \| --- \| --- \| --- \| --- \| --- \| --- \| --- \| --- \| --- \| --- \| --- \| --- \| --- \| --- \| --- \| --- \| --- \| --- \| --- \| --- \| --- \| --- \| --- \| --- \| --- \| --- \| --- \| --- \| --- \| --- \| --- \| --- \| --- \| --- \| --- \| --- \| --- \| --- \| --- \| --- \| --- \| --- \| --- \| --- \| --- \| --- \| --- \| --- \| --- \| --- \| --- \| --- \| --- \| --- \| --- \| --- \| --- \| --- \| --- \| --- \| --- \| --- \| --- \| --- \| --- \| --- \| --- \| --- \| --- \| --- \| --- \| --- \| --- \| --- \| --- \| --- \| --- \| --- \| --- \| --- \| --- \| --- \| --- \| --- \| --- \| --- \| --- \| --- \| --- \| --- \| --- \| --- \| --- \| --- \| --- \| --- \| --- \| --- \| --- \| --- \| --- \| --- \| --- \| --- \| --- \| --- \| --- \| --- \| --- \| --- \| --- \| --- \| --- \| --- \| --- \| --- \| --- \| --- \| --- \| --- \| --- \| --- \| --- \| --- \| --- \| --- \| --- \| --- \| --- \| --- \| --- \| --- \| --- \| --- \| --- \| --- \| --- \| --- \| --- \| --- \| --- \| --- \| --- \| --- \| --- \| --- \| --- \| --- \| --- \| --- \| --- \| --- \| --- \| --- \| --- \| --- \| --- \| --- \| --- \| --- \| --- \| --- \| --- \| --- \| --- \| --- \| --- \| --- \| --- \| --- \| --- \| --- \| --- \| --- \| --- \| --- \| --- \| --- \| --- \| --- \| --- \| --- \| --- \| --- \| --- \| --- \| --- \| --- \| --- \| --- \| --- \| --- \| --- \| --- \| --- \| --- \| --- \| --- \| --- \| --- \| --- \| --- \| --- \| --- \| --- \| --- \| --- \| --- \| --- \| --- \| --- \| --- \| --- \| --- \| --- \| --- \| --- \| --- \| --- \| --- \| --- \| --- \| --- \| --- \| --- \| --- \| --- \| --- \| --- \| --- \| --- \| --- \| --- \| --- \| --- \| --- \| --- \| --- \| --- \| --- \| --- \| --- \| --- \| --- \| --- \| --- \| --- \| --- \| --- \| --- \| --- \| --- \| --- \| --- \| --- \| --- \| --- \| --- \| --- \| --- \| --- \| --- \| --- \| --- \| --- \| --- \| --- \| --- \| --- \| --- \| --- \| --- \| --- \| --- \| --- \| --- \| --- \| --- \| --- \| --- \| --- \| --- \| --- \| --- \| --- \| --- \| --- \| --- \| --- \| --- \| --- \| --- \| --- \| --- \| --- \| --- \| --- \| --- \| --- \| --- \| --- \| --- \| --- \| --- \| --- \| --- \| --- \| --- \| --- \| --- \| --- \| --- \| --- \| --- \| --- \| --- \| --- \| --- \| --- \| --- \| --- \| --- \| --- \| --- \| --- \| --- \| --- \| --- \| --- \| --- \| --- \| --- \| --- \| --- \| --- \| --- \| --- \| --- \| --- \| --- \| --- \| --- \| --- \| --- \| --- \| --- \| --- \| --- \| |
| --- | --- | --- | --- | --- | --- | --- | --- | --- | --- | --- | --- | --- | --- | --- | --- | --- | --- | --- | --- | --- | --- | --- | --- | --- | --- | --- | --- | --- | --- | --- | --- | --- | --- | --- | --- | --- | --- | --- | --- | --- | --- | --- | --- | --- | --- | --- | --- | --- | --- | --- | --- | --- | --- | --- | --- | --- | --- | --- | --- | --- | --- | --- | --- | --- | --- | --- | --- | --- | --- | --- | --- | --- | --- | --- | --- | --- | --- | --- | --- | --- | --- | --- | --- | --- | --- | --- | --- | --- | --- | --- | --- | --- | --- | --- | --- | --- | --- | --- | --- | --- | --- | --- | --- | --- | --- | --- | --- | --- | --- | --- | --- | --- | --- | --- | --- | --- | --- | --- | --- | --- | --- | --- | --- | --- | --- | --- | --- | --- | --- | --- | --- | --- | --- | --- | --- | --- | --- | --- | --- | --- | --- | --- | --- | --- | --- | --- | --- | --- | --- | --- | --- | --- | --- | --- | --- | --- | --- | --- | --- | --- | --- | --- | --- | --- | --- | --- | --- | --- | --- | --- | --- | --- | --- | --- | --- | --- | --- | --- | --- | --- | --- | --- | --- | --- | --- | --- | --- | --- | --- | --- | --- | --- | --- | --- | --- | --- | --- | --- | --- | --- | --- | --- | --- | --- | --- | --- | --- | --- | --- | --- | --- | --- | --- | --- | --- | --- | --- | --- | --- | --- | --- | --- | --- | --- | --- | --- | --- | --- | --- | --- | --- | --- | --- | --- | --- | --- | --- | --- | --- | --- | --- | --- | --- | --- | --- | --- | --- | --- | --- | --- | --- | --- | --- | --- | --- | --- | --- | --- | --- | --- | --- | --- | --- | --- | --- | --- | --- | --- | --- | --- | --- | --- | --- | --- | --- | --- | --- | --- | --- | --- | --- | --- | --- | --- | --- | --- | --- | --- | --- | --- | --- | --- | --- | --- | --- | --- | --- | --- | --- | --- | --- | --- | --- | --- | --- | --- | --- | --- | --- | --- | --- | --- | --- | --- | --- | --- | --- | --- | --- | --- | --- | --- | --- | --- | --- | --- | --- | --- | --- | --- | --- | --- | --- | --- | --- | --- | --- | --- | --- | --- | --- | --- | --- | --- | --- | --- | --- | --- | --- | --- | --- | --- | --- | --- | --- | --- | --- | --- | --- | --- | --- | --- | --- | --- | --- | --- | --- | --- | --- | --- | --- | --- | --- | --- | --- | --- | --- | --- | --- | --- | --- | --- | --- | --- | --- | --- | --- | --- | --- | --- | --- | --- | --- | --- | --- | --- | --- | --- | --- | --- | --- | --- | --- | --- | --- | --- | --- | --- | --- | --- | --- | --- | --- | --- | --- | --- | --- | --- | --- | --- | --- | --- | --- | --- | --- | --- | --- | --- | --- | --- | --- | --- | --- | --- | --- | --- | --- | --- | --- | --- | --- | --- | --- | --- | --- | --- | --- | --- | --- | --- | --- | --- | --- | --- | --- | --- | --- | --- | --- | --- | --- | --- | --- | --- | --- | --- | --- | --- | --- | --- | --- | --- | --- | --- | --- | --- | --- | --- | --- | --- | --- | --- | --- | --- | --- | --- | --- | --- | --- | --- | --- | --- | --- | --- | --- | --- | --- | --- | --- | --- | --- | --- | --- | --- | --- | --- | --- | --- | --- | --- | --- | --- | --- | --- | --- | --- | --- | --- | --- | --- | --- | --- | --- | --- | --- | --- | --- | --- | --- | --- | --- | --- | --- | --- | --- | --- | --- | --- | --- | --- | --- | --- | --- | --- | --- | --- | --- | --- | --- | --- | --- | --- | --- | --- | --- | --- | --- | --- | --- | --- | --- |
